# Alisertib impairs the stemness of hepatocellular carcinoma by inhibiting purine synthesis

**DOI:** 10.1016/j.jbc.2025.108558

**Published:** 2025-04-30

**Authors:** Zhuoran Qi, Jie Luo, Wenfeng Liu, Ye Xu, Yifan Ma, Sunkuan Hu, Xizhong Shen, Xiaojing Du, Wei Xiang

**Affiliations:** 1 Department of Gastroenterology and Hepatology, Zhongshan Hospital, Fudan University, Shanghai, China; 2 Shanghai Institute of Liver Diseases, Zhongshan Hospital, Fudan University, Shanghai, China; 3 Huashan Hospital, Fudan University, Shanghai, China; 4 Department of Gastroenterology, The First Affiliated Hospital of Wenzhou Medical University, Wenzhou, China; 5 Endoscopy Center, Department of Gastroenterology, Shanghai East Hospital, School of Medicine, Tongji University, Shanghai, China; 6 Department of Interventional Radiology, The First Affiliated Hospital of Wenzhou Medical University, Wenzhou, China

**Keywords:** hepatocellular carcinoma, tumor-repopulating cells, purine metabolism, alisertib, AURKA–AKT

## Abstract

Hepatocellular carcinoma tumor–repopulating cells (HCC-TRCs) drive disease progression, yet their purine metabolism mechanisms remain poorly understood. This study revealed that the stemness index, strongly linked to poor HCC prognosis, exhibited a robust positive correlation with purine metabolism through single-sample gene set enrichment analysis. Integrated drug screening across CTRP, GDSC, and PRISM databases identified alisertib, an aurora kinase A (AURKA) inhibitor, as a potent agent targeting stemness. Using fibrin gel–based 3D-cultured HCC-TRCs, mechanistic studies demonstrated that alisertib suppresses xanthine and hypoxanthine production by inhibiting the AURKA–AKT signaling axis. This disruption markedly impaired tumor spheroid formation, migration, and invasion *in vitro*, while significantly suppressed tumor growth *in vivo*, which could be rescued by the AKT agonist SC79. Our findings revealed a novel therapeutic strategy targeting purine metabolism through AURKA–AKT axis inhibition, effectively eliminating HCC-TRCs.

Hepatocellular carcinoma (HCC) represents approximately 90% of primary liver cancer and remains a major cause of cancer mortality and medical problem worldwide (1). HCC exhibits significant intratumoral heterogeneity, driving differential therapeutic sensitivity and poor prognosis. Such biological complexity is driven by tumor-repopulating cells (TRCs), a kind of cancer stem–like cells (CSLCs), possessing self-renewal capacity and playing an important role in cancer progression, recurrence, and metastasis (2, 3). TRCs further demonstrate intrinsic resistance to conventional anticancer therapy (4), contributing to treatment failure in HCC, underscoring the urgency to identify HCC-TRCs–specific vulnerabilities.

Metabolic reprogramming is a predominant hallmark of cancer and offers promising therapeutic targeting opportunities (5). Emerging evidence reveals TRCs-related metabolic adaptations, including impaired oxidative phosphorylation (6), increased sphingolipid metabolism (7), glycogen metabolic program activation (8) and kynurenine metabolism dominance (9). Based on these specific changes, the life cycle of TRCs is heavily dependent on metabolism alternations to acquire and maintain stemness. Despite metabolic targeting advances, little has been reported about purine metabolic alterations in TRCs, which was considered as a crucial driver for HCC progression (10).

In this article, single-sample gene set enrichment analysis (ssGSEA) was used to calculate the prognosis-related stemness index. This ssGSEA stemness index was significantly associated with several metabolic processes, especially the purine biosynthesis. Through integrative analysis, alisertib—a drug targeting aurora kinase A (AURKA)—was screened for TRCs as an effective drug in HCC. Mechanistic studies revealed that alisertib inhibited the activation of AURKA–AKT signaling and decreased the level of xanthine and hypoxanthine in HCC-TRCs, establishing its potential as a purine metabolism–targeted strategy to overcome HCC stemness.

## Results

### Purine anabolism as a potential driver of HCC stemness

**Figure 1:**
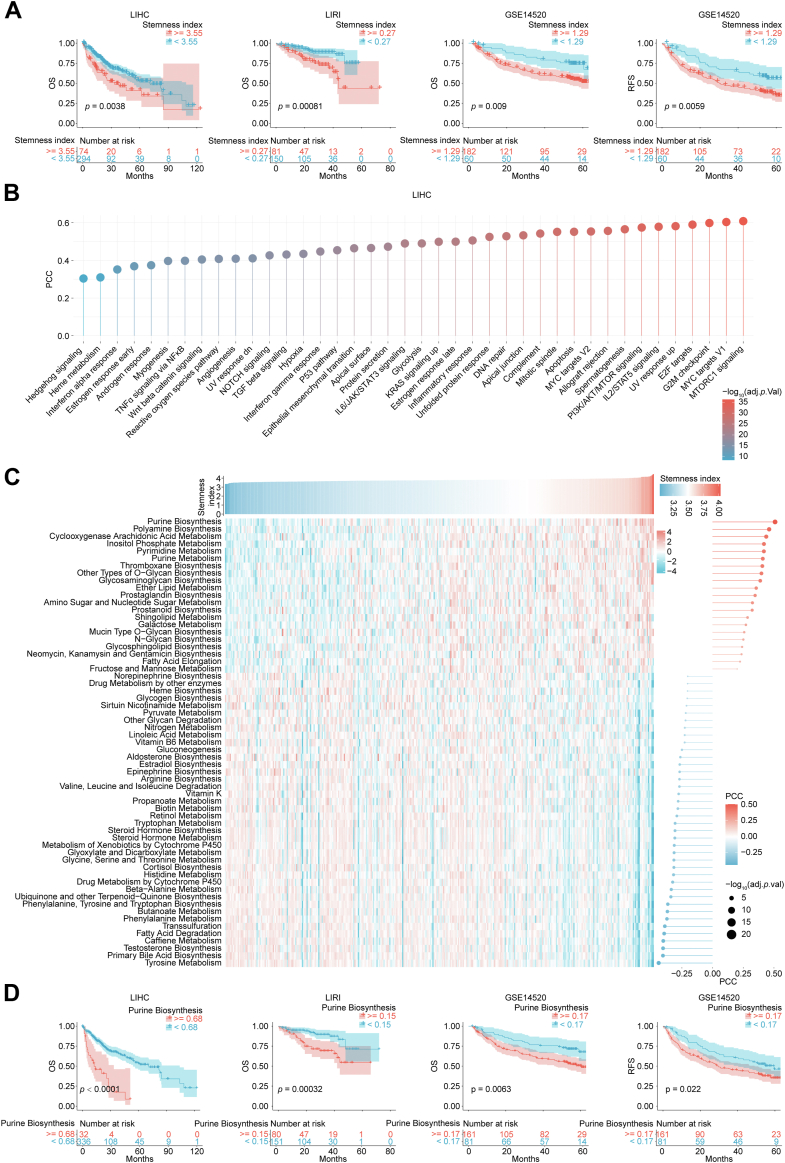
**Purine biosynthesis was positive correlation to stemness index in HCC.***A,* survival analysis of stemness index in LIHC, LIRI, and GSE14520 cohort (log-rank test). *B,* the results of GSVA showed that majority of cancer hallmarks enriched in patients with high stemness index. *C,* correlation between stemness index and multiple metabolism processes determined by ssGSEA (Pearson correlation analysis). *D,* relationship between purine biosynthesis and prognosis in LIHC, LIRI, and GSE14520 cohort (log-rank test). GSVA, gene set variation analysis; HCC, hepatocellular carcinoma; OS, overall survival; PCC, Pearson correlation coefficient; RFS, relapse-free survival; ssGSEA, single-sample gene set enrichment analysis.

To explore the stemness mechanism, ssGSEA was employed to calculate the stemness index of each HCC sample from LIHC, LIRI, and GSE14520 datasets (Table S1). Survival analysis revealed significantly poorer prognosis in patients with high stemness indices across all cohorts. In detail, HCC patients with high stemness index exhibited a shorter overall survival (OS) than those with low stemness index in LIHC (median OS [mOS] 33.5 *versus* 70.5 months, *p* = 0.0038), LIRI (mOS 44.0 months *versus* not reached, *p* = 0.00081), and GSE14520 (mOS not reached, *p* = 0.009) cohort, as well a shorter median relapse–free survival (30.1 months *versus* not reached, *p* = 0.0059) in GSE14520 cohort (Fig. 1*A*). The results of gene set variation analysis (GSVA) indicated that majority of cancer hallmarks were enriched in patients with high stemness index (Fig. 1*B*), accounting for its poor prognosis. Metabolism reprogramming is a fundamental hallmark of cancer (5). Previous studies demonstrated that TRCs displayed a better metabolic adaptability and thereby could change their metabolic preferences to better adapt to intricate tumor microenvironment (11). Consistently, the stemness index was significantly associated with multiple metabolism processes, among which the most relevant one was purine biosynthesis (Pearson correlation coefficient [PCC] = 0.51, adjacent *p* < 0.0001, Fig. 1*C* and Table S2). In addition, survival analysis showed that patients with high score of purine biosynthesis had a poorer prognosis than those with low score in LIHC (mOS 12.4 *versus* 71.0 months, *p* < 0.0001), LIRI (mOS not reached, *p* = 0.00032), and GSE14520 (mOS 60.5 months *versus* not reached, *p* = 0.0063; median relapse–free survival 29.9 *versus* 59.5 months, *p* = 0.022) cohort (Fig. 1*D*). Taken together, these results suggested that enhanced purine biosynthesis may be a driving factor in HCC *via* regulating stemness.

### Targeting AURKA is a promising treatment for overcoming stemness in HCC

**Figure 2:**
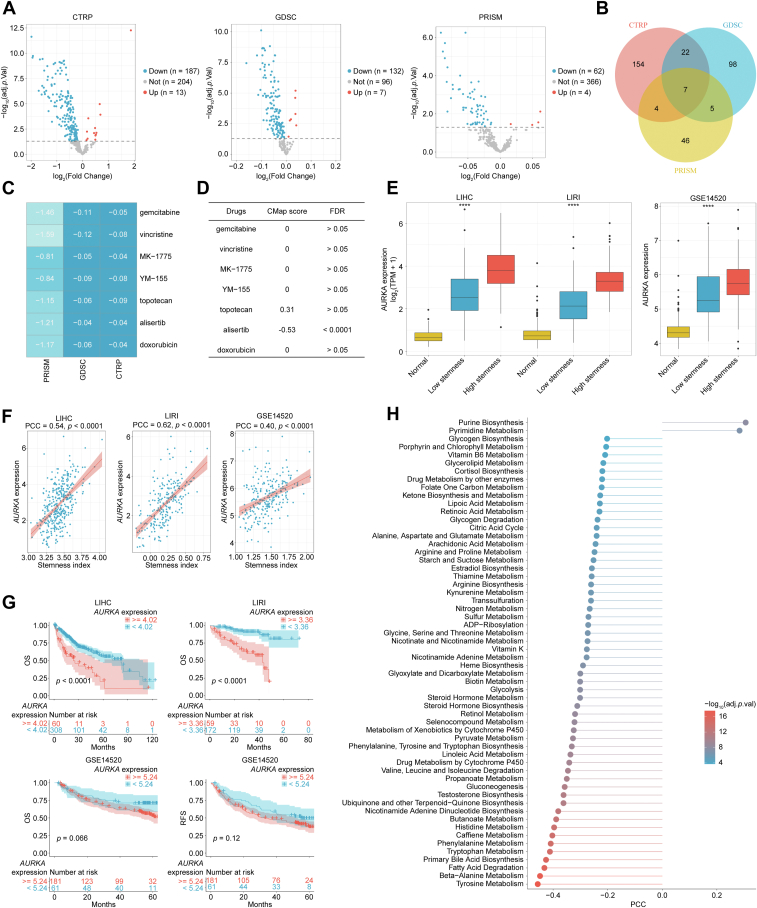
**The screening process of alisertib and relationship of *AURKA* to prognosis and metabolism.***A,* numerous candidate drugs (*blue*) were selected in CTRP, GDSC, and PRISM databases (cutoff value, adjacent *p* < 0.05). *B,* Venn diagram showed the number of common drugs in all three databases. *C,* the fold change of sensitivity of seven common drugs in three databases. *D,* the CMap score and FDR of seven drugs screened out. *E, AURKA* expression in normal, low stemness, and high stemness HCC in LIHC, LIRI, and GSE14520 databases (Kruskal–Wallis test). *F,* the correlation between AURKA expression and stemness index in LIHC, LIRI, and GSE14520 databases (Pearson correlation analysis). *G,* the survival analysis AURKA in LIHC, LIRI, and GSE14520 cohort (log-rank test). *H,* correlation of *AURKA* with various metabolic processes (Pearson correlation analysis). ∗∗∗∗*p* < 0.0001. AURKA, aurora kinase A; CMap, connectivity map; FDR, false discovery rate; PCC, Pearson correlation coefficient.

To explore drugs for overcoming stemness, human cancer cell lines (CCLs) transcriptomic data were downloaded from CCLE database, and then the stemness index of each cell line was calculated by ssGSEA algorithm. The sensitivity data of each cell line to different treatments were further obtained from CTRP, GDSC, and PRISM databases. According to the value of stemness index, all the CCLs were divided into tertiles, with the upper and lower tertiles designated as high and low stemness groups, respectively. Then, the different drug sensitivity between high and low stemness CCLs was analyzed by using limma package. A total of 187, 132, and 64 candidate drugs were selected in CTRP, GDSC, and PRISM databases, respectively (adjacent *p* < 0.05; Fig. 2*A*). Among them, gemcitabine, vincristine, MK-1775, YM-155, topotecan, alisertib, and doxorubicin were identified by all three databases (Fig. 2, *B* and *C*). To further confirm the specific drugs, the differentially expressed genes (DEGs) between low and high stemness HCC were also identified by limma package. Setting |logFC| >1 and adjacent *p* < 0.05 as threshold, a total of 456, 1022, and 707 DEGs were identified in LIHC, LIRI, and GSE14520 datasets, respectively (Fig. S1*A*). After intersecting, 91 overlapped DEGs were confirmed among these three databases (Fig. S1*B*). Then, *MAGEA6*, *CCL19*, *OLFML3*, and *BGN* were excluded because of their inconsistent trends across datasets (Fig. S1*C*). Finally, 87 DEGs were uploaded to the connectivity map (CMap) website, and the CMap score of these seven candidate drugs was calculated. As shown, alisertib (CMap score = −0.53; false discovery rate <0.0001) was the most likely drug to overcome stemness in HCC (Fig. 2*D*).

Alisertib is a well-known drug targeting AURKA, an evolutionarily highly conserved serine/threonine kinase. *AURKA* was significantly upregulated in HCC, and its expression in high stemness HCC was higher than in low stemness HCC as well as in normal tissues (Fig. 2*E*). The expression of *AURKA* was significantly positive correlation to stemness index in LIHC (PCC = 0.54, *p* < 0.0001), LIRI (PCC = 0.62, *p* < 0.0001), GSE14520 (PCC = 0.40, *p* < 0.0001) cohort, as well in CCLs (PCC = 0.20, *p* < 0.0001; Figs. 2*F* and S2). Survival analyses showed that high *AURKA* HCC displayed a poorer outcome than low *AURKA* HCC in LIHC (mOS 23.7 *versus* 81.9 months, *p* < 0.0001) and LIRI (mOS 43.0 months *versus* not reached, *p* < 0.0001) cohort, with a consistent trend in GSE14520 cohort (Fig. 2*G*).

**Figure 3:**
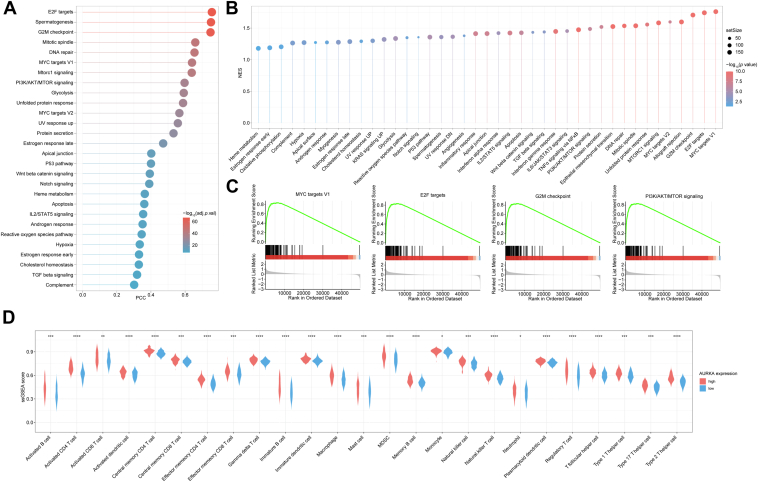
***AURKA* was closely associated with majority of cancer hallmark signatures.***A*, GSVA of different signaling pathways in HCC. *B,* GSEA of different signaling pathways in HCC. *C,* GSEA with *AURKA* peaks for “MYC target V1,” “E2F targets,” “G2M checkpoint,” and “PI3K–AKT–MTOR signaling” signatures. *D,* the ssGSEA score of various types of immune cells in high and low *AURKA* expression groups (unpaired *t* test or Mann–Whitney test). ∗*p* < 0.05, ∗∗*p* < 0.01, ∗∗∗*p* < 0.001, ∗∗∗∗*p* < 0.0001. AURKA, aurora kinase A; GSEA, gene set enrichment analysis; GSVA, gene set variation analysis; HCC, hepatocellular carcinoma; NES, normalized enrichment score; ssGSEA, single-sample gene set enrichment analysis.

Of note, *AURKA* was negative correlation with most metabolism processes but positive correlation with purine biosynthesis and pyrimidine metabolism (Fig. 2*H*). The results of GSVA and GSEA demonstrated that majority of cancer hallmark signatures were significantly enriched in high *AURKA* HCC, such as PI3K–AKT–MTOR signaling (12) (a classical downstream cascade of AURKA; Fig. 3, *A*–*C*). In addition, the expression of *AURKA* was significantly associated with immune infiltration in HCC, and more immune cells and stromal cells infiltrated in tumor with high *AURKA* (Fig. 3*D*). Taken together, targeting AURKA may be a promising strategy for overcoming stemness in HCC.

### AURKA–AKT signaling and purine biosynthesis were active in HCC-TRCs

**Figure 4:**
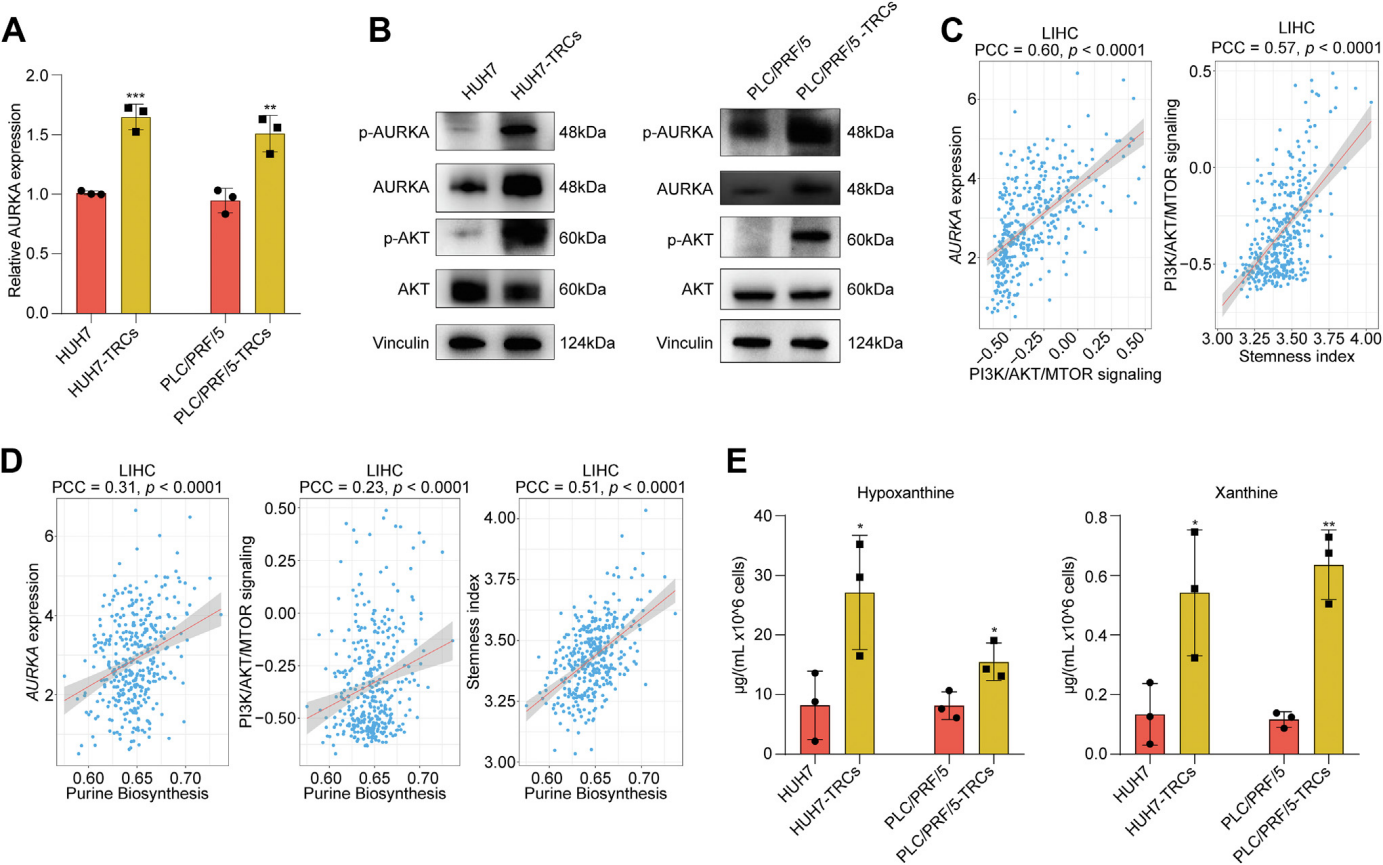
**AURKA–AKT signaling and purine biosynthesis was promoted in HCC-TRCs**. *A* and *B*, the mRNA expression of *AURKA* (*A*, unpaired *t* test, n = 3) and protein level of p-AURKA, AURKA, p-AKT, and AKT (*B*) in 2D-cultured HCC cell and HCC-TRCs. *C,* correlation analysis between PI3K–AKT–MTOR signaling and *AURKA* expression or stemness index (Pearson correlation analysis). *D,* correlation analysis between purine biosynthesis and *AURKA* expression, PI3K–AKT–MTOR signaling, and stemness index. *E,* content of hypoxanthine (*left*) and xanthine (*right*) in 2D-cultured HCC cell and HCC-TRCs (unpaired *t* test, n = 3). Results represent three independent experiments and are displayed as mean ± SD, ∗*p* < 0.05, ∗∗*p* < 0.01, and ∗∗∗*p* < 0.001. AURKA, aurora kinase A; HCC-TRCs, hepatocellular carcinoma tumor–repopulating cells.

HCC-TRCs, a subpopulation with well-documented stemness properties (13), exhibited elevated AURKA expression compared with their parental 2D–cultured HCC counterparts, as confirmed by quantitative RT–PCR and Western blotting (Fig. 4, *A* and *B*). Notably, HCC-TRCs also demonstrated increased phosphorylation level of AURKA, indicating a more active AURKA signaling (Fig. 4*B*). AKT signaling pathway has been convinced as the downstream of AURKA (12). We found that AURKA was positively related to PI3K–AKT–MTOR signaling, and the latter was also positive correlation with stemness in HCC (Fig. 4*C*). Consistently, a higher phosphorylation level of AKT was observed in HCC-TRCs when compared with their parental 2D HCC cells (Fig. 4*B*), collectively suggesting activation of the AURKA–AKT signaling cascade in stemness-enriched HCC-TRCs. Furthermore, AURKA and PI3K–AKT–MTOR signaling pathway were positively related to purine metabolism (Fig. 4*D*), a pathway that itself correlated positively with stemness index (Fig. 4*D*). Compared with HUH7 and PLC/PRF/5 cells, we detected a significant elevation of xanthine and hypoxanthine in HCC-TRCs (Fig. 4*E*), reinforcing the link between AURKA–AKT signaling activation and enhanced purine metabolism in this stemness-associated subpopulation.

### Alisertib effectively repressed HCC-TRCs *via* blocking AURKA–AKT signaling

**Figure 5:**
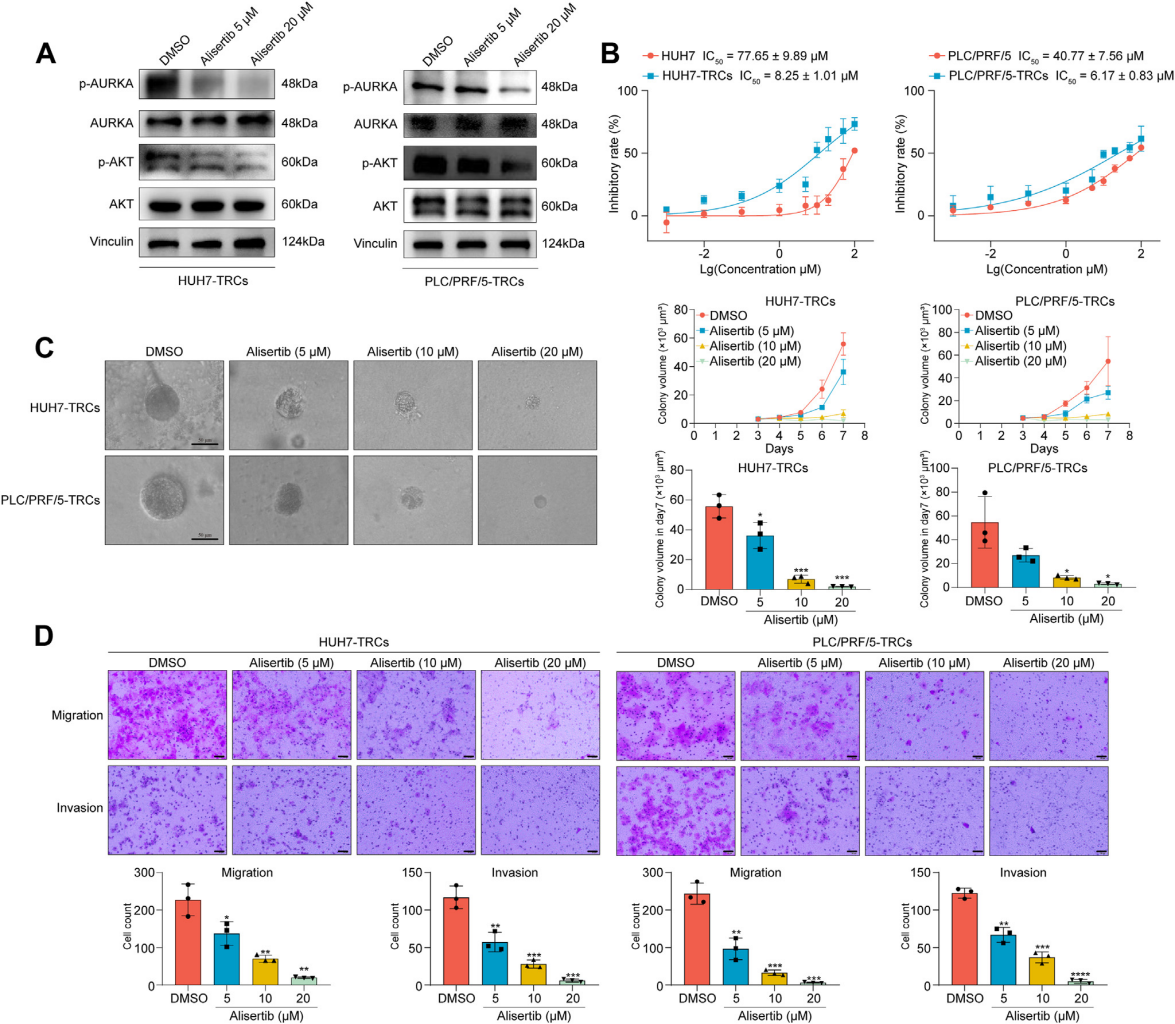
**Alisertib inhibited the growth, migration, and invasion of HCC-TRCs.***A,* alisertib decreased the phosphorylation of AURKA and AKT (48 h). *B,* the inhibitory effect of alisertib on HCC-TRCs and 2D cells was detected using CCK8 assay (48 h, n = 3). *C*, effect of alisertib on the colony growth of HCC-TRCs (n = 3, Tukey multiple comparisons test). Scale represents 50 μm. *D,* effect of alisertib on the migration and invasion ability of HCC-TRCs (48 h, n = 3, Tukey multiple comparisons test). Scale represents 50 μm. Results represent three independent experiments and are displayed as mean ± SD, ∗*p* < 0.05, ∗∗*p* < 0.01, ∗∗∗*p* < 0.001, and ∗∗∗∗*p* < 0.0001. AURKA, aurora kinase A; HCC-TRCs, hepatocellular carcinoma tumor–repopulating cells.

Based on the discoveries of AURKA–AKT signaling pathway, HUH7-TRCs and PLC/PRF/5-TRCs were treated with alisertib, which was found to be the most HCC-TRCs–targeted drug (Fig. 2*D*). Consistent with expectations, the alisertib showed concentration-dependent inhibition of AURKA and AKT phosphorylation (Fig. 5*A*). The results of Cell Counting Kit 8 assay showed that alisertib exerted a more potent inhibition against HCC-TRCs than 2D cells. In particularly, the IC_50_ of alisertib was reduced by 89.37% in HUH7-TRCs (8.25 ± 1.01 μM *versus* 77.65 ± 9.89 μM) and 84.87% in PLC/PRF/5-TRCs (6.17 ± 0.83 μM *versus* 40.77 ± 7.56 μM) than parental cells, respectively. Furthermore, alisertib dose-dependently attenuated HCC-TRCs phenotypes, including colony spheroid formation (Fig. 5*C*), migration, and invasion ability (Fig. 5*D*) of HUH7-TRCs and PLC/PRF/5-TRCs. These data demonstrated that suppressing AURKA–AKT signaling is a promising strategy to selectively kill HCC-TRCs.

### Alisertib reduced purine biosynthesis *via* AURKA–AKT signaling

**Figure 6:**
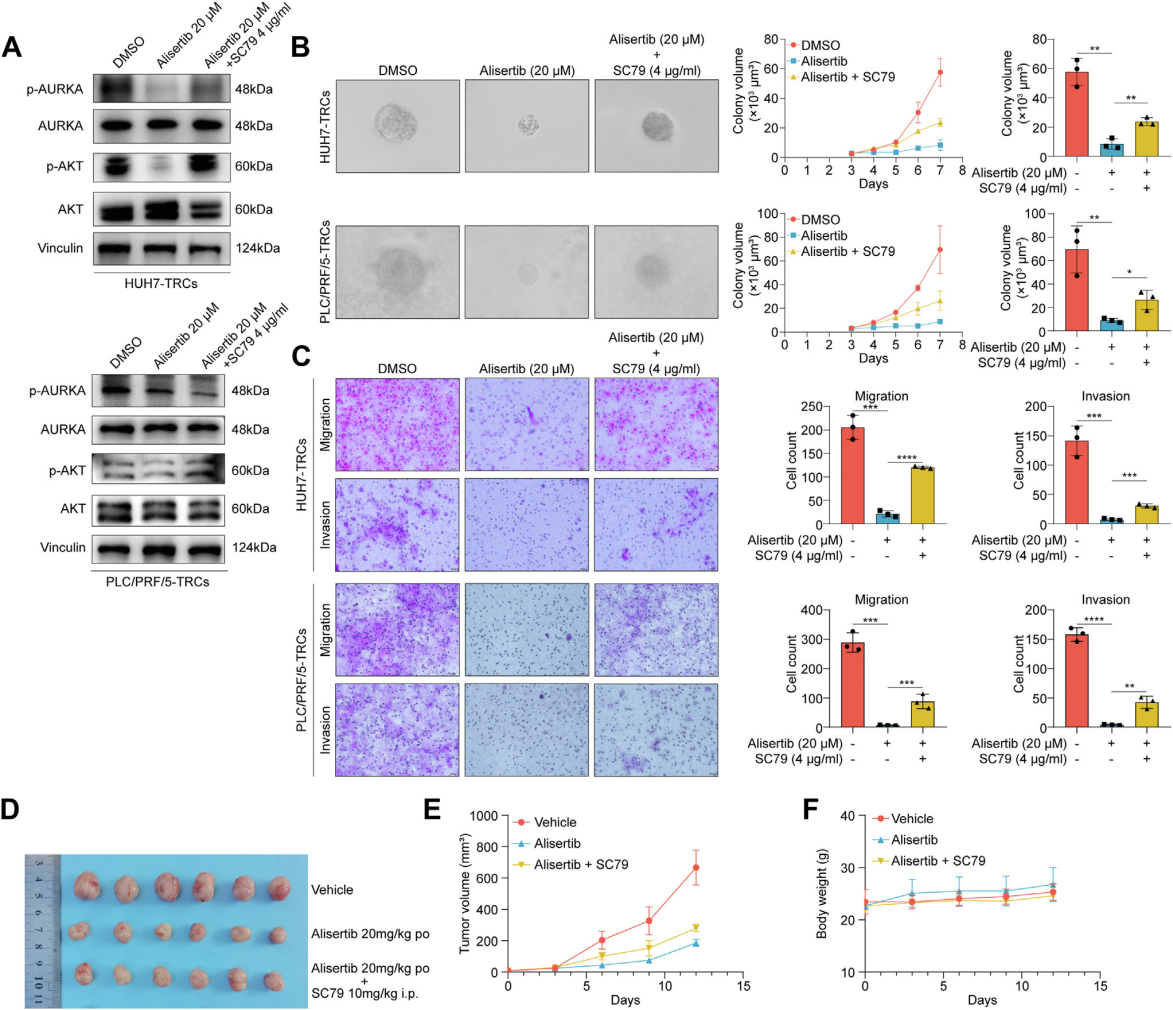
**SC79 rescued the inhibition of alisertib on HCC-TRCs.***A,* the protein levels of p-AURKA and p-AKT under alisertib and SC79 (48 h). *B,* effect of alisertib and SC79 on the colony growth of HCC-TRCs (n = 3, Tukey multiple comparisons test). Scale represents 50 μm. *C*, effect of alisertib and SC79 on the migration and invasion ability of HCC-TRCs (48 h, n = 3, Tukey multiple comparisons test). Scale represents 50 μm. *D*–*F,* inhibitory effects of alisertib on the growth of xenograft tumor of PLC/PRF/5-TRCs subcutaneously transplanted to the flanks of BALB/c nude mice (n = 6). After tumor grew to 50 mm^3^, mice were treated with alisertib (20 mg/kg, po, qd) compared with alisertib (20 mg/kg, po, qd) + SC79 (10 mg/kg, i.p., qd) and the carrier. The isolated tumor (*D*), tumor volume (*E*), and body weight (*F*) were recorded. Results represent three independent experiments and are displayed as mean ± SD, ∗*p* < 0.05, ∗∗*p* < 0.01, ∗∗∗*p* < 0.001, and ∗∗∗∗*p* < 0.0001. AURKA, aurora kinase A; HCC-TRCs, hepatocellular carcinoma tumor–repopulating cells.

**Figure 7:**
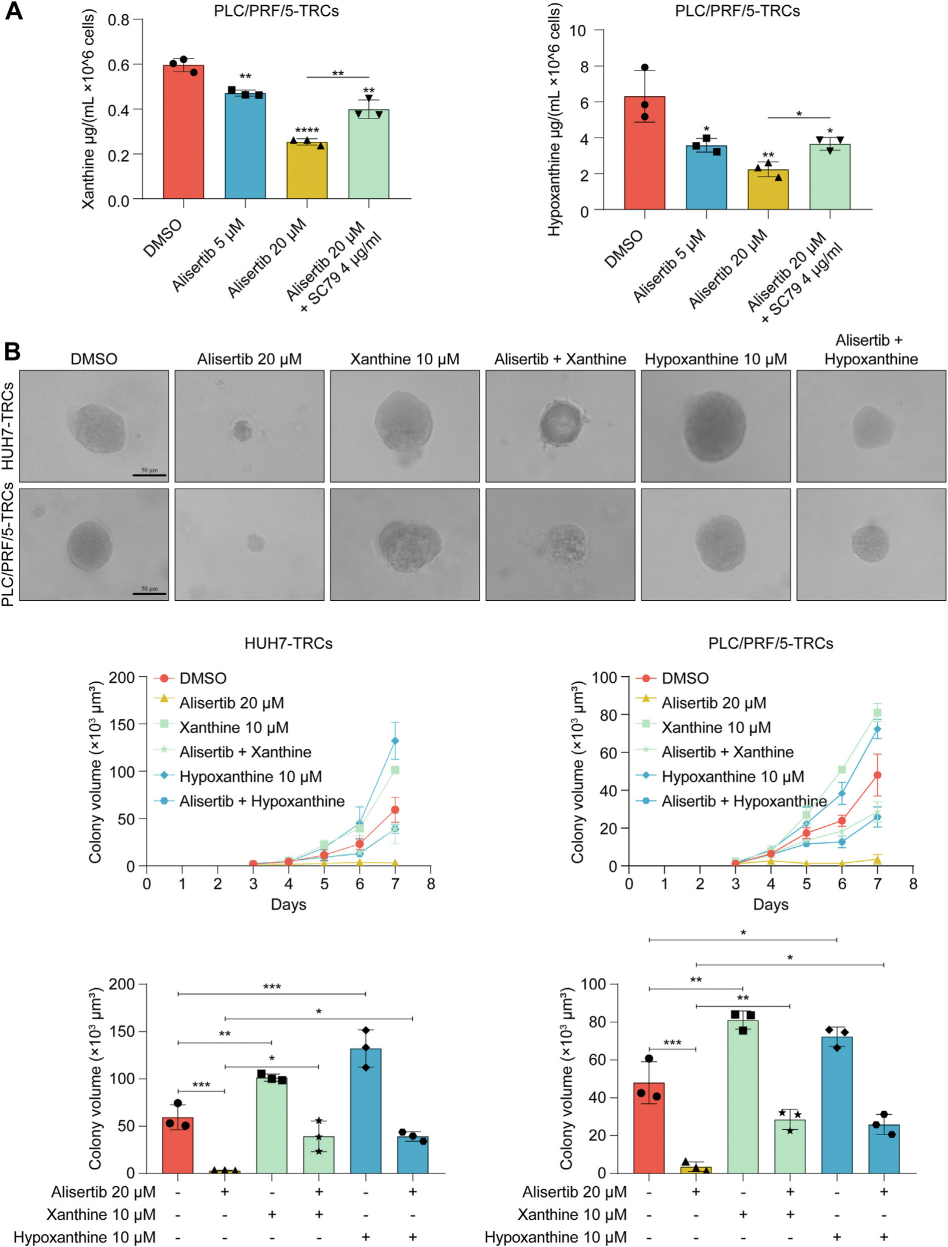
**SC79 rescued the inhibition of alisertib on purine biosynthesis.***A,* content of hypoxanthine and xanthine when alisertib and SC79 added (48 h, n = 3, Tukey multiple comparisons test). *B,* effect of exogenous hypoxanthine and xanthine on the colony growth of HCC-TRCs and the inhibitory effect of alisertib (n = 3, Tukey multiple comparisons test). Scale represents 50 μm. Results represent three independent experiments and are displayed as mean ± SD, ∗*p* < 0.05, ∗∗*p* < 0.01, and ∗∗∗*p* < 0.001. HCC-TRCs, hepatocellular carcinoma tumor–repopulating cells.

To validate the underlying mechanisms, we performed rescue experiments. In both HUH7-TRCs and PLC/PRF/5-TRCs, SC79 (AKT agonist) restored AKT phosphorylation without reversing alisertib-induced suppression of AURKA phosphorylation (Fig. 6*A*). Functionally, SC79 rescued the inhibition of alisertib on the colony spheroids, migration, and invasion ability in both HUH7-TRCs and PLC/PRF/5-TRCs (Fig. 6, *B* and *C*). *In vivo* pharmacological estimation showed that alisertib significantly decreased the growth of PLC/PRF/5-TRCs–formed tumor in mice, an effect partially abrogated upon SC79 cotreatment (Fig. 6, *D* and *E*). Notably, these treatments have not caused significant change of body weight (Fig. 6*F*). Given the regulatory role of purine, we detected four kinds of purines in 2D and 3D HCC cells. HPLC analysis identified distinct purine metabolism patterns in HCC-TRCs, demonstrating significant elevation in xanthine and hypoxanthine concentrations, respectively, compared with parental cells. While guanine levels showed no significant variation, adenine fell below the detection threshold in both cell populations (Figs. 7*A* and S3). Subsequent supplementation with exogenous xanthine or hypoxanthine not only promoted spheroid growth but also attenuated alisertib’s therapeutic efficacy (Fig. 7*B*). Of note, alisertib dose-dependently decreased xanthine and hypoxanthine in PLC/PRF/5-TRCs, which also could be rescued by SC79 (Fig. 7*A*). These data indicated that alisertib inhibited HCC-TRCs *via* suppressing AURKA–AKT signaling–mediated purine biosynthesis.

## Discussion

Integrated bioinformatics analysis of LIHC, LIRI, and GSE14520 datasets revealed a positive correlation between purine biosynthesis and HCC stemness index, with elevated purine biosynthesis predicting poorer prognosis. Stratifying cell lines into high/low stemness groups using CCLE-derived index identified alisertib as the most promising therapeutic candidate for attenuating stemness through comprehensive drug sensitivity profiling. Elevated AURKA expression was associated with enhanced stemness, immune infiltration, AKT signaling pathway activation, purine metabolism, and poorer prognosis. Mechanistically, alisertib effectively suppressed HCC-TRCs and decreased purine biosynthesis *via* blocking AURKA–AKT signaling.

As an oncogene, *AURKA* overexpressed in a variety of tumors and plays multiple roles in cancer development (12). *AURKA* is highly expressed in CSLCs of various tumors, including HCC, breast cancer, ovarian cancer, colorectal cancer, and acute myelocytic leukemia (14). It regulates CSLCs both in the cytoplasm and nucleus in chronic myelogenous leukemia (15) and colorectal cancer (16). Alisertib is the first oral selective AURKA inhibitor with demonstrated clinical efficacy in phase II/III clinical trials for relapsed or refractory peripheral T-cell lymphoma (NCT01482962), ovarian cancer (NCT00853307), and acute myelocytic leukemia (NCT02560025), showing translational potential for HCC. Our findings align with prior evidence of AKT phosphorylation activation in CSLCs (17, 18), confirming the capacity of alisertib to inhibit AURKA–AKT and reduce HCC-TRC stemness.

In recent years, numerous articles have investigated the mechanisms underlying stemness generation in HCC. However, limited attention has been given to the role of purine metabolism in this process. Xanthine dehydrogenase/oxidase, a key enzyme catalyzing the final two steps of purine catabolism, has been associated with HCC pathogenesis. HCC cancer stem cells demonstrate xanthine dehydrogenase/oxidase deficiency, with this enzyme loss potentiating the expansion of liver cancer stem cells (19). Xanthine dehydrogenase, a rate-limiting enzyme involved in purine metabolism, was associated with the expression of cancer stem biomarkers, such as CD44 or CD133 in HCC (20). These findings align with our observations, as both existing articles and our works emphasize the critical role of increased purine biosynthesis in maintaining HCC stemness. Our study specifically identifies AURKA–AKT signaling as an upstream regulator of hypoxanthine and xanthine biosynthesis in HCC-TRCs. AKT signaling exerts multifaceted metabolic effects *via* either direct control of metabolic enzymes and nutrient transporters or transcriptional regulation of metabolic pathway components (21). These effects include regulation of glucose uptake and glycolysis, control of anabolic metabolism (such as *de novo* lipid synthesis and protein synthesis), and redox homeostasis (21). Given that *de novo* purine synthesis requires coordinated input from multiple metabolic pathways, AKT signaling likely regulates it through multiple parallel mechanisms affecting these metabolic inputs (21). Hence, we inferred that AURKA–AKT signaling may boost hypoxanthine and xanthine biosynthesis through an intricate influence on other cellular metabolisms in HCC-TRCs. And investigation of the mechanisms *via* which AKT signaling regulates purine biosynthesis is of great importance to understanding HCC-TRCs.

In summary, our study establishes a significant connection between stemness, enhanced purine biosynthesis, and poor prognosis in HCC. We also provided a novel agent to inhibit purine biosynthesis of HCC-TRCs *via* suppressing AURKA–AKT signaling. Further study of alisertib could pave the way for developing novel therapeutic strategies to combat HCC progression by counteracting cancer stemness mechanisms.

## Experimental procedures

### Bioinformatics analyses

HCC RNA-Seq count data were downloaded from The Cancer Genome Atlas (https://portal.gdc.cancer.gov) and International Cancer Genome Consortium (www.icgc.org) databases and transformed into transcripts per million. The Cancer Genome Atlas (LIHC, n = 368) and IGGC (LIRI, n = 231) samples containing complete prognostic information were included in the subsequent analysis. HCC microarray data (GSE14520) were from Gene Expression Omnibus (https://www.ncbi.nlm.nih.gov/geo/). The microarray comprised two cohorts, GSE14520-GPL571 and GSE14520-GPL3921, which were merged (n = 242) and batch-corrected using the sva package. Gene expression data of human cell lines were downloaded from the CCLE database (22). CTRP, GDSC, and PRISM were three large databases that include data on the varying sensitivities of different human CCLs to different drugs. Sensitivity was expressed as the area under the curve value, where a lower area under the curve value indicated higher sensitivity (23, 24). After obtaining the drug sensitivity data, those with a not available value of more than 20% were deleted and corrected using the neighbor method (K-Nearest Neighbor) to fill in remaining not available values.

A stemness gene set (n = 189) was obtained from Miranda *et al.* (25), and stemness index for each sample was calculated using ssGSEA (26). The optimal cutoff value was calculated using survminer package together with patients’ prognostic information. Accordingly, patients were categorized into high or low stemness groups, and the survival analysis was performed using the Kaplan–Meier method. A set of 114 metabolism-related genes was obtained from the study by Rosario *et al.*, and metabolic scores of each samples were calculated using ssGSEA (25, 26). In addition, the “h.all.v7.5.1.symbols” gene set was downloaded from MsigDB data (https://www.gsea-msigdb.org/gsea/msigdb), and tumor characteristic scores were calculated using the GSVA algorithm (28). Differential genes between patients with high and low expression of AURKA and the “h.all.v7.5.1.symbols” gene set was used for GSEA (26, 27).

CMap (https://clue.io/) analysis enabled the calculation of a standardized score (−100 to 100) for each intervention. A negative score indicated that the intervention resulted in a gene expression opposite to the disease gene expression, suggesting potential therapeutic value. Therefore, we used the limma package to calculate DEGs between high and low stemness tumors and uploaded the DEGs to the CMap website to calculate CMap scores.

### Cell culture and reagents

Human HCC cell lines, HUH7 and PLC/PRF/5, were from the Liver Cancer Institute, Zhongshan Hospital, Fudan University, maintained in Dulbecco's modified Eagle's medium (DMEM; 41401ES76; Yeasen Biotechnology Co, Ltd) with 10% fetal bovine serum (Sigma) and 1% penicillin–streptomycin (60162ES76; Yeasen), and kept at 37 °C in 5% CO_2_ in a humidified ThermoForma incubator (Thermo Fisher Scientific). All cell lines have been authenticated using short tandem repeat.

Alisertib (HY-10971), SC79 (HY-18749), xanthine (HY-W017389), and hypoxanthine (HY-N0091) were purchased from MedChemExpress. Aurora A (T55522S) antibody (Ab), Phospho-Aurora A (Thr288) Ab (TA3011S), AKT1/2/3 (T55561) Ab, and Phospho-Akt (Ser473) Ab (T40067) were from Abmart Shanghai Co, Ltd. Vinculin (A23468) was sourced from ABclonal Technology Co, Ltd. Rabbit horseradish peroxidase–conjugated secondary Ab (A0208) were from Beyotime Biotechnology. Salmon fibrinogen (SEA-133) and thrombin (SEA-135) were purchased from Sea Run Holdings, Inc.

### Isolation of HCC-TRCs using fibrin gel–based 3D culture system

Fibrin gel–based 3D culture system was established to isolate HCC-TRCs, by forming environment with 90 Pa pressure, which was optimal for cancer cell spheroid formation (13). Cells cultured in 2D rigid plates were trypsinized and resuspended with complete medium first and then mixed by an isochoric salmon fibrinogen (2 mg ml^−1^) diluted with T7 buffer (50 mm Tris–HCl, 150 mm NaCl, pH 7.4). Meanwhile, 100 U ml^−1^ thrombin was added to culture plate and mixed with the cell suspension (volume, thrombin/cell suspension  1:50). After incubation for 30 min in 37 °C humidified incubator, the fibrin gel–based 3D culture system was shaped, and then complete medium was added to each well.

### Cell Counting Kit 8

HCC-TRCs or 2D cell lines were treated with a serious of gradient concentration alisertib for 48 h. Then, Cell Counting Kit 8 was added and incubated for 4 h at 37 °C, 5% CO_2_ incubator. The absorbance value was detected at 450 nm using FlexStation 3 multimode microplate reader (Molecular Devices).

### Quantitative RT–PCR

Total RNA was extracted from HCC cells in 2D/3D cultures using RNA-Quick Purification Kit (ES-RN001; TISHAN Biotechnology Co, Ltd). Complementary DNA was obtained using Hifair Ⅲ 1st Strand Complementary DNA Synthesis SuperMix (11141ES; Yeasen Biotechnology Co, Ltd). Then quantitative RT–PCR was performed using SYBR Green kit (11202ES08; Yeasen) on an ABI Prism 7500 sequence detection system (Applied Biosystems). The conditions were set as follows: initial denaturation at 95 °C for 5 min, 40  cycles of 95 °C for 10 s and 60 °C for 30 s. The 2^−ΔΔCT^ method was used to calculate the gene expression change, with β-ACTIN as the internal normalization. The primes were:

β-ACTIN forward primer 5′-CATGTACGTTGCTATCCAGGC-3′

β-ACTIN reverse primer 5′-CTCCTTAATGTCACGCACGAT-3′

AURKA forward primer 5′-GAGGTCCAAAACGTGTTCTCG-3′

AURKA reverse primer 5′-ACAGGATGAGGTACACTGGTTG-3’.

### Western blotting

Total proteins were obtained from HCC cells in 2D/3D cultures applying radioimmunoprecipitation assay lysis buffer (P0013B; Beyotime Biotechnology) and separated on 10% SDS-PAGE and then transferred to polyvinylidene difluoride (ISEQ00010; Merck Millipore Ltd) membranes. After blocked by Western blocking buffer (WB6017; WellBio Technology Co, Ltd) for 1 h, the membranes were probed with the primary Ab at 4 °C overnight and then incubated with corresponding secondary Ab for 1 h. Finally, the bands were detected by Chemistar High-sig ECL Western Blotting Substrate (180-5001; Tanon) and visualized by Odyssey Imaging System (LiCor Biosciences).

### Transwell assay

Invasion and migration ability of HCC-TRCs was evaluated by transwell assay. In detail, 50 μl Matrigel (356234; BD Biosciences) diluted at 1:8 with DMEM was added to the microporous membrane of Transwell Permeable Supports (3422; Corning, Inc) in order to investigate the invasion ability. After incubated at 37 °C for 4 h, 500 μl of DMEM containing 20% fetal bovine serum was added to the lower chamber and 300 μl of DMEM with 3 × 10^4^ pretreated cells to the upper chamber and cultured at 37 °C for 48 h. The supports were taken out, and the cells above the microporous membrane were removed by cotton swab carefully. The cells passing through the membrane filter were stained with 0.1% crystal violet solution (V5265; Sigma). The invasive cells were recorded using microscope and counted by ImageJ software (National Institutes of Health). For the investigation on migration ability, all the processes were consistent to the aforementioned besides no Matrigel used.

### HPLC

Pretreated cells were collected and mixed with 0.2 ml of 5% perchloric acid aqueous solution. After vortexed for 1 min, the samples were subjected to ultrasonic extraction under low-temperature conditions for 60 min. The mixture was then centrifuged at 12,000 rpm for 30 min. The resulting supernatant was filtered through a 0.22 μm microporous membrane filter prior to instrumental analysis. The level of purines were detected using the Agilent 1200 HPLC system. The conditions were set as follows: detector, VWD; column: Agilent 5 TC-C18 column (250 nm × 4.6 nm × 5 μm); column temperature: 30 °C; wavelength: 254 nm; flow rate, 1.0 ml/min; injection volume: 20 μl; and mobile phase: methanol:acetonitrile:phosphate = 49:20:31. Then, the concentration of purine was calculated according to the peak area–concentration standard curve.

### *In vivo* estimation

Male BALB/c nude mice (4–6 weeks old) were purchased from Beijing Vital River Laboratory Animal Technology Co, Ltd for *in vivo* estimation. PLC/PRF/5-TRCs (5 × 10^6^) were subcutaneously injected to the right scapula of mice. When the tumor grew to 50 mm^3^, mice were randomly divided into three groups according to the weight: (1) vehicle group; (2) alisertib group: oral administration (po) of alisertib 20 mg/kg; and (3) alisertib + SC79 group: alisertib 20 mg/kg po and intraperitoneal injection (i.p.) of SC79 10 mg/kg. The body weight, long diameter (L), and short diameter (W) of tumor were measured every 3 days. The volume (V) of tumor was calculated as follows, V = 0.5 × L × W^2^. At the end, mice were euthanized and the tumor was separated. All murine experiments were approved by the Institutional Animal Care and Use Committee at Zhongshan Hospital, Fudan University (2021-014).

### Statistical analysis

R software (version 4.3.0; R Core Team, R Foundation for Statistical Computing) and GraphPad Prism 9 (GraphPad Software, Inc) were employed for statistical analyses and data visualization. The Student’s *t* test and Mann–Whitney test were applied for continuous data with normally and non-normally distributions, respectively. OS represented the time from diagnosis to the last follow-up or death, and recurrence-free survival referred to the duration from complete remission post-therapy to tumor recurrence or study endpoint. For cellular experiments, Student’s *t* test was used to compare the difference between two groups, and Tukey multiple comparisons test was used for pairwise comparisons among multiple groups. The data were displayed as mean ± SD of three independent experiments. A significance threshold of *p* < 0.05 was adopted unless otherwise mentioned.

## Data availability

Data sources and handling of the publicly available datasets used in this article are described in the “Bioinformatics analyses” section.

## Supporting information

This article contains supporting information.

## Conflict of interest

The authors declare that they have no conflicts of interest with the contents of this article.

## Supporting information

Supplmentary Figures

Supplmentary Tables
