# Supplementary material for: Alisertib impairs the stemness of hepatocellular carcinoma by inhibiting purine synthesis
Source: J Biol Chem. 2025 Apr 30;301(6):108558. doi: 10.1016/j.jbc.2025.108558 (PMC12152889; doi:10.1016/j.jbc.2025.108558)
Supplement: Supplmentary Figures [file mmc1.docx]

**
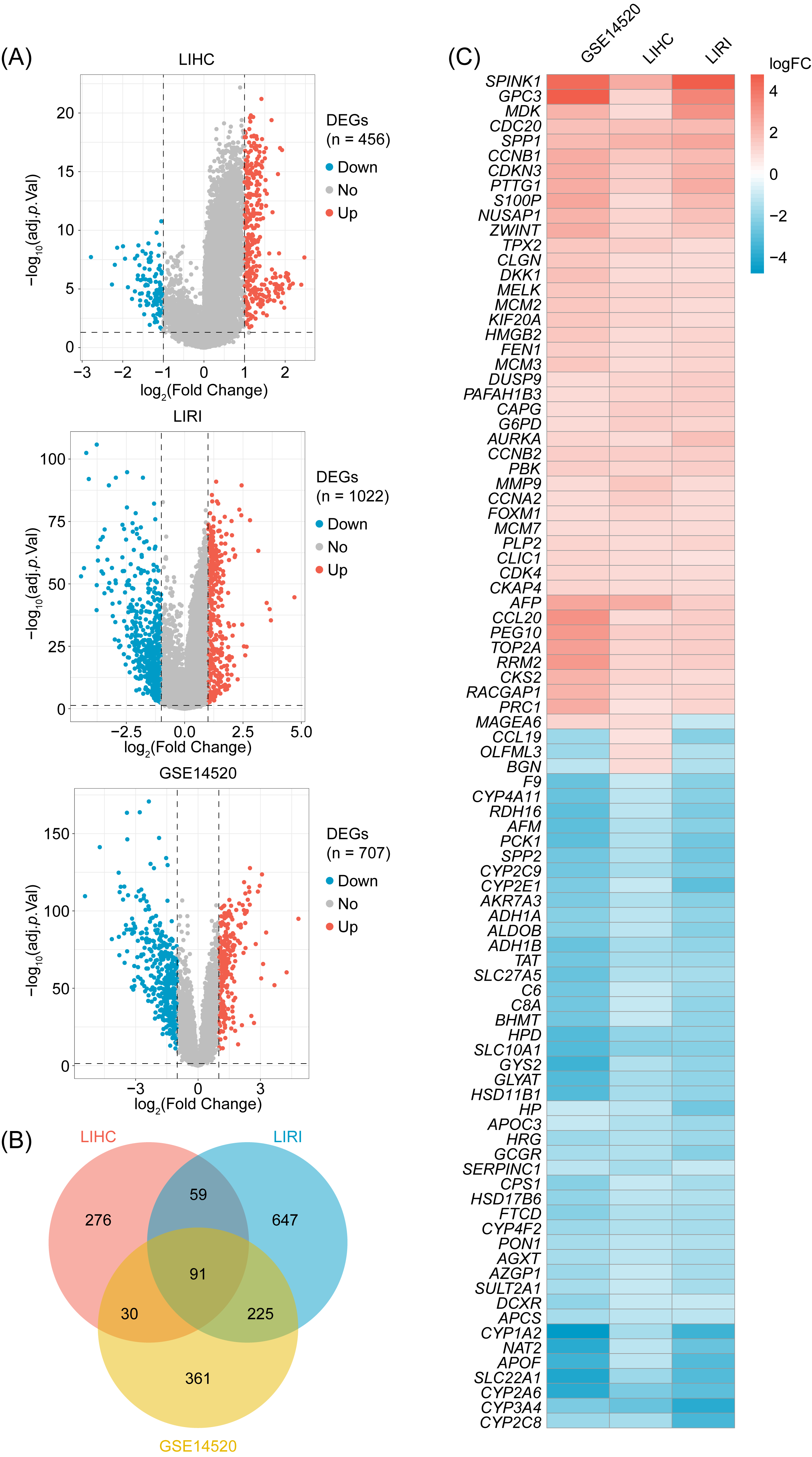
**

**Figure S1.** The DEGs in low and high stemness groups of HCC. (A) The DEGs between low and high stemness HCC in LIHC, LIRI and GSE14520 databases. (|logFC > 1| and adj.*p*.val < 0.05) (B) Venn diagram showed the number of common genes in all three databases. (C) The expression trends of DEGs in three databases. DEGs, differentially expressed genes; LogFC, Log_2_(Fold Change).


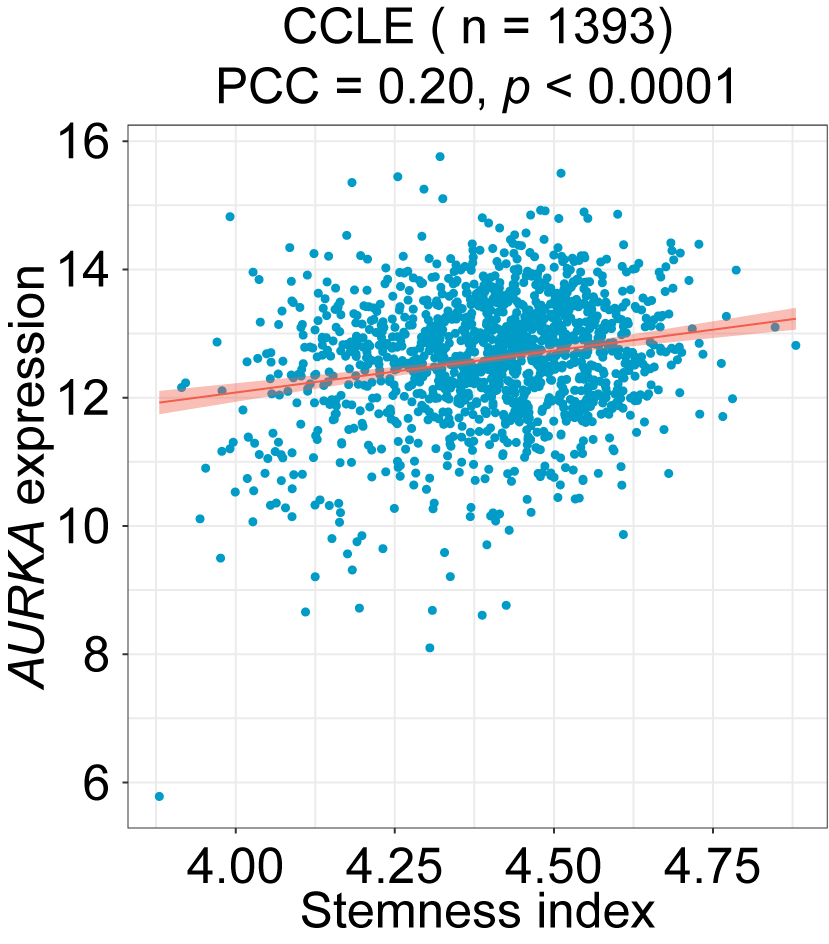


**Figure S2.** The correlation between *AURKA* expression and stemness index in CCLs.


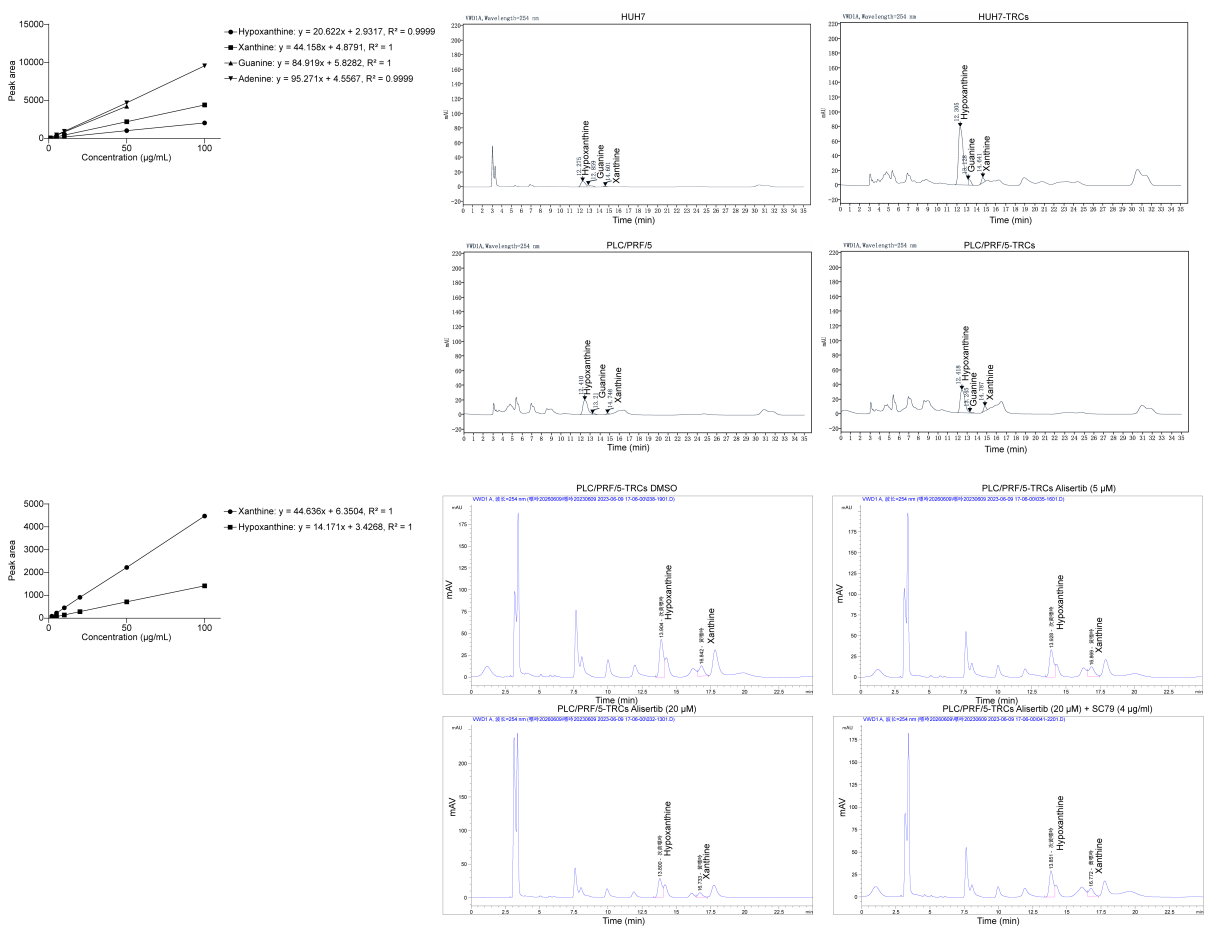


**Figure S3.** The chromatogram of HPLC and peak area-concentration standard curve.


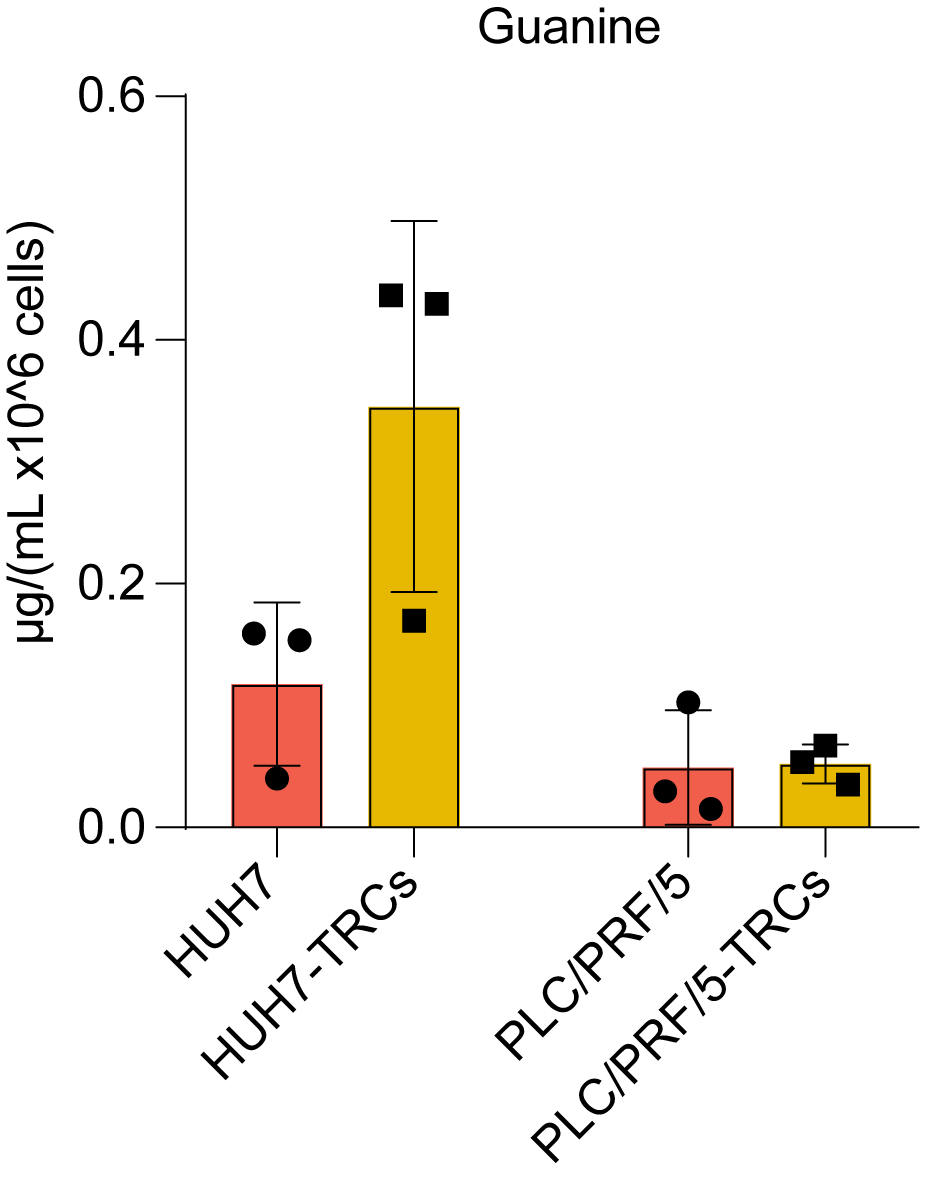


**Figure S4** The level of guanine between HCC-TRCs and 2D cells.
