# Supplementary material for: Alisertib impairs the stemness of hepatocellular carcinoma by inhibiting purine synthesis
Source: J Biol Chem. 2025 Apr 30;301(6):108558. doi: 10.1016/j.jbc.2025.108558 (PMC12152889; doi:10.1016/j.jbc.2025.108558)
Supplement: Supplmentary Tables [file mmc2.docx]

| **Table S1 Stemness index, purine buosynthesis score and AURKA expression in each HCC cohort** | | | | |
| --- | --- | --- | --- | --- |
| **ID** | **HCC cohort** | **Stemness index** | **Purine biosynthesis** | **AURKA expression** |
| GSM363448 | GSE14520 | 1.292261499 | 0.108789668 | 5.591086653 |
| GSM363126 | GSE14520 | 1.47137919 | 0.116518205 | 5.442542006 |
| GSM363010 | GSE14520 | 1.272288304 | 0.1210615 | 5.057412723 |
| GSM363122 | GSE14520 | 1.250526998 | 0.124267188 | 5.841496442 |
| GSM363055 | GSE14520 | 1.227259425 | 0.126873737 | 4.623895402 |
| GSM363440 | GSE14520 | 1.184428121 | 0.134418348 | 5.957697426 |
| GSM363143 | GSE14520 | 1.403533138 | 0.137056786 | 5.637081669 |
| GSM363424 | GSE14520 | 1.158823779 | 0.138291805 | 6.104993335 |
| GSM363289 | GSE14520 | 1.100883275 | 0.13980062 | 7.364567215 |
| GSM362977 | GSE14520 | 1.206910129 | 0.140073633 | 5.156163821 |
| GSM362950 | GSE14520 | 1.322482813 | 0.140079834 | 5.239530537 |
| GSM362971 | GSE14520 | 1.049329063 | 0.14015324 | 4.944177801 |
| GSM363269 | GSE14520 | 1.237250486 | 0.141951771 | 5.97020171 |
| GSM363314 | GSE14520 | 1.718111495 | 0.143618065 | 6.11108694 |
| GSM363035 | GSE14520 | 1.134433594 | 0.143893344 | 5.028774905 |
| GSM363436 | GSE14520 | 1.416433352 | 0.14474565 | 6.773112304 |
| GSM363107 | GSE14520 | 1.353700649 | 0.145556578 | 5.977114287 |
| GSM363230 | GSE14520 | 1.270833771 | 0.147664992 | 6.803660978 |
| GSM363200 | GSE14520 | 1.278794322 | 0.148396936 | 4.873077011 |
| GSM363241 | GSE14520 | 1.350835295 | 0.149504114 | 5.880667052 |
| GSM362978 | GSE14520 | 1.266707959 | 0.149628319 | 5.904038475 |
| GSM363205 | GSE14520 | 1.491011 | 0.14967216 | 6.063027743 |
| GSM363295 | GSE14520 | 1.06716153 | 0.149778341 | 4.575177536 |
| GSM363076 | GSE14520 | 1.309117431 | 0.150010823 | 4.703225122 |
| GSM363016 | GSE14520 | 1.133573186 | 0.151485819 | 4.555757145 |
| GSM363243 | GSE14520 | 1.172585894 | 0.151659541 | 4.908627406 |
| GSM363124 | GSE14520 | 1.117752655 | 0.15177736 | 4.867810615 |
| GSM363084 | GSE14520 | 1.188390112 | 0.152061109 | 4.960965488 |
| GSM363393 | GSE14520 | 1.496742458 | 0.152566954 | 3.844749238 |
| GSM363315 | GSE14520 | 1.234520825 | 0.152616336 | 5.170976486 |
| GSM363344 | GSE14520 | 1.252237418 | 0.152645417 | 5.135754932 |
| GSM363039 | GSE14520 | 1.278548348 | 0.152661683 | 5.995876996 |
| GSM362976 | GSE14520 | 1.16535661 | 0.152929358 | 4.426722047 |
| GSM363360 | GSE14520 | 1.476322869 | 0.152999795 | 5.327660903 |
| GSM363056 | GSE14520 | 1.246651788 | 0.153081778 | 6.404377701 |
| GSM363087 | GSE14520 | 1.420376618 | 0.154367149 | 4.551477272 |
| GSM363082 | GSE14520 | 1.261327177 | 0.154543842 | 5.594618697 |
| GSM363293 | GSE14520 | 1.322452452 | 0.156924342 | 4.669320907 |
| GSM363071 | GSE14520 | 1.303181469 | 0.1570741 | 5.282235398 |
| GSM363275 | GSE14520 | 1.138277863 | 0.15815649 | 5.183155459 |
| GSM363444 | GSE14520 | 1.283766718 | 0.158233083 | 4.925409045 |
| GSM363108 | GSE14520 | 1.389111659 | 0.158271021 | 4.927390115 |
| GSM363336 | GSE14520 | 1.353628074 | 0.15844162 | 5.577501181 |
| GSM362994 | GSE14520 | 1.142621898 | 0.158446591 | 5.212122118 |
| GSM363333 | GSE14520 | 1.312316168 | 0.159048018 | 4.782226 |
| GSM363294 | GSE14520 | 1.549430667 | 0.159149096 | 5.874741986 |
| GSM363188 | GSE14520 | 1.452071468 | 0.15967934 | 5.329307084 |
| GSM362993 | GSE14520 | 1.464648161 | 0.159805079 | 5.396786672 |
| GSM363388 | GSE14520 | 1.731791462 | 0.159981572 | 4.987957126 |
| GSM363164 | GSE14520 | 1.159125028 | 0.160242727 | 5.063337789 |
| GSM363290 | GSE14520 | 1.470203115 | 0.160324174 | 5.30396064 |
| GSM363186 | GSE14520 | 1.165188667 | 0.160338075 | 4.057392941 |
| GSM363192 | GSE14520 | 1.28260172 | 0.160560394 | 6.581142166 |
| GSM363312 | GSE14520 | 1.155093798 | 0.160738118 | 5.105800761 |
| GSM363335 | GSE14520 | 1.224273843 | 0.16083923 | 4.109731022 |
| GSM363168 | GSE14520 | 1.20351444 | 0.161666041 | 4.479060129 |
| GSM362972 | GSE14520 | 1.119470824 | 0.161751196 | 6.086069336 |
| GSM363109 | GSE14520 | 1.439467078 | 0.1625514 | 5.880996881 |
| GSM363378 | GSE14520 | 1.513449545 | 0.163289254 | 6.066319117 |
| GSM363176 | GSE14520 | 1.18108891 | 0.163862163 | 4.507039278 |
| GSM363128 | GSE14520 | 1.209657326 | 0.164365494 | 5.289147975 |
| GSM363270 | GSE14520 | 1.432491905 | 0.164441104 | 6.910970834 |
| GSM363144 | GSE14520 | 1.383047572 | 0.165278994 | 5.195993101 |
| GSM363272 | GSE14520 | 1.315304124 | 0.16545702 | 4.047846672 |
| GSM363202 | GSE14520 | 1.312494019 | 0.165466771 | 5.675264768 |
| GSM362952 | GSE14520 | 1.260385948 | 0.165536201 | 4.751258155 |
| GSM362966 | GSE14520 | 1.258750698 | 0.165704079 | 6.087056847 |
| GSM363102 | GSE14520 | 1.212240553 | 0.165847992 | 5.004415971 |
| GSM363054 | GSE14520 | 1.45985224 | 0.166569819 | 4.404996806 |
| GSM363352 | GSE14520 | 1.556988729 | 0.16715815 | 5.175584212 |
| GSM362947 | GSE14520 | 1.462463035 | 0.167525036 | 6.144055125 |
| GSM363291 | GSE14520 | 1.538294929 | 0.167786274 | 5.667035839 |
| GSM363074 | GSE14520 | 1.20288791 | 0.168229877 | 5.21541448 |
| GSM363098 | GSE14520 | 1.412717625 | 0.168363921 | 4.709479029 |
| GSM363014 | GSE14520 | 1.31081688 | 0.168908587 | 5.468217291 |
| GSM363339 | GSE14520 | 1.602809437 | 0.168910885 | 5.196980612 |
| GSM363224 | GSE14520 | 1.283522294 | 0.168919248 | 7.49985622 |
| GSM363182 | GSE14520 | 1.153098469 | 0.16896278 | 6.120632221 |
| GSM363292 | GSE14520 | 1.471304582 | 0.169562553 | 6.911299675 |
| GSM363343 | GSE14520 | 1.281925009 | 0.169647811 | 5.453404626 |
| GSM362949 | GSE14520 | 1.431559959 | 0.169999511 | 5.341661251 |
| GSM363125 | GSE14520 | 1.442406738 | 0.170841387 | 5.267093892 |
| GSM363366 | GSE14520 | 1.263023208 | 0.170846505 | 5.292440336 |
| GSM363029 | GSE14520 | 1.472678832 | 0.171072386 | 5.909304871 |
| GSM363081 | GSE14520 | 1.317084108 | 0.17129206 | 6.17988288 |
| GSM363438 | GSE14520 | 1.459215353 | 0.171469207 | 4.986443093 |
| GSM363072 | GSE14520 | 1.603427039 | 0.171859729 | 5.702586235 |
| GSM363288 | GSE14520 | 1.374200622 | 0.172033351 | 5.564663538 |
| GSM363368 | GSE14520 | 1.363553347 | 0.173399543 | 5.059387745 |
| GSM363073 | GSE14520 | 1.384917318 | 0.173948609 | 5.328319573 |
| GSM363264 | GSE14520 | 1.248900764 | 0.174126181 | 5.378682633 |
| GSM363036 | GSE14520 | 1.358232318 | 0.174205494 | 6.4425608 |
| GSM363099 | GSE14520 | 1.443204542 | 0.174330989 | 5.160771547 |
| GSM363327 | GSE14520 | 1.284429143 | 0.174978488 | 5.574868477 |
| GSM363273 | GSE14520 | 1.220808746 | 0.175291708 | 4.564644744 |
| GSM363428 | GSE14520 | 1.460259607 | 0.175421785 | 4.705686473 |
| GSM363142 | GSE14520 | 1.225469931 | 0.175524697 | 5.540634434 |
| GSM362948 | GSE14520 | 1.472591795 | 0.175875011 | 5.805520014 |
| GSM363151 | GSE14520 | 1.486584862 | 0.176027955 | 6.288838916 |
| GSM363148 | GSE14520 | 1.511982907 | 0.176169349 | 5.834254036 |
| GSM363011 | GSE14520 | 1.450005523 | 0.176203137 | 5.672302235 |
| GSM363217 | GSE14520 | 1.145160161 | 0.176482904 | 4.723962853 |
| GSM363220 | GSE14520 | 1.467549197 | 0.176596842 | 5.345765929 |
| GSM363121 | GSE14520 | 1.572720863 | 0.176675736 | 5.415220539 |
| GSM363331 | GSE14520 | 1.665344117 | 0.176977092 | 5.9906106 |
| GSM363326 | GSE14520 | 1.575797405 | 0.17713107 | 5.802653685 |
| GSM362987 | GSE14520 | 1.339106289 | 0.177459883 | 5.414233028 |
| GSM363130 | GSE14520 | 1.09501148 | 0.177581258 | 5.109092135 |
| GSM363050 | GSE14520 | 1.28924992 | 0.17762877 | 5.972835402 |
| GSM362984 | GSE14520 | 1.335535246 | 0.178030773 | 4.914223631 |
| GSM363430 | GSE14520 | 1.659634125 | 0.178056124 | 6.194509531 |
| GSM363355 | GSE14520 | 1.417662529 | 0.178152029 | 5.424436979 |
| GSM362965 | GSE14520 | 1.457277661 | 0.178259562 | 6.607475134 |
| GSM363169 | GSE14520 | 1.529303626 | 0.178289214 | 5.850712882 |
| GSM363033 | GSE14520 | 1.826431354 | 0.178609544 | 6.156841286 |
| GSM363123 | GSE14520 | 1.310739153 | 0.178909713 | 5.811541284 |
| GSM363251 | GSE14520 | 1.253939896 | 0.179136862 | 5.325028199 |
| GSM363152 | GSE14520 | 1.306627028 | 0.179560576 | 6.720051386 |
| GSM363012 | GSE14520 | 1.136472882 | 0.179653176 | 4.527448167 |
| GSM363215 | GSE14520 | 1.51543679 | 0.179862039 | 5.564993367 |
| GSM363364 | GSE14520 | 1.340728996 | 0.179952536 | 4.784529864 |
| GSM363101 | GSE14520 | 1.409843465 | 0.180338113 | 5.478750083 |
| GSM363358 | GSE14520 | 1.371905175 | 0.180417087 | 5.229897316 |
| GSM363008 | GSE14520 | 1.344426307 | 0.180463072 | 5.455379648 |
| GSM363106 | GSE14520 | 1.633995645 | 0.180606175 | 5.922142514 |
| GSM363127 | GSE14520 | 1.704719924 | 0.181129368 | 5.715423877 |
| GSM363313 | GSE14520 | 1.291119597 | 0.181317853 | 4.936277713 |
| GSM363328 | GSE14520 | 1.312869172 | 0.181906721 | 5.70752379 |
| GSM363048 | GSE14520 | 1.523006501 | 0.182070127 | 5.640044202 |
| GSM363267 | GSE14520 | 1.416852116 | 0.182711725 | 5.245697492 |
| GSM363265 | GSE14520 | 1.505119007 | 0.183103041 | 4.883939631 |
| GSM362982 | GSE14520 | 1.37523665 | 0.183261499 | 5.552813407 |
| GSM363247 | GSE14520 | 1.236177694 | 0.18357876 | 5.578488692 |
| GSM362964 | GSE14520 | 1.253546223 | 0.183775019 | 5.610418872 |
| GSM363239 | GSE14520 | 1.480893421 | 0.183790393 | 5.579147362 |
| GSM362988 | GSE14520 | 1.418624297 | 0.183802379 | 5.796728619 |
| GSM362954 | GSE14520 | 1.530242317 | 0.183859093 | 5.552839056 |
| GSM363037 | GSE14520 | 1.576145022 | 0.184134078 | 6.280609 |
| GSM363178 | GSE14520 | 1.322518115 | 0.184276575 | 6.331630729 |
| GSM363034 | GSE14520 | 1.44312469 | 0.184472418 | 6.415240322 |
| GSM363145 | GSE14520 | 2.036983031 | 0.184500438 | 7.335929397 |
| GSM363266 | GSE14520 | 1.402733982 | 0.184512535 | 6.171982792 |
| GSM363400 | GSE14520 | 1.48771135 | 0.184578379 | 6.288509087 |
| GSM363362 | GSE14520 | 1.437878812 | 0.184681586 | 5.160771547 |
| GSM363316 | GSE14520 | 1.644423054 | 0.186468677 | 5.882313233 |
| GSM363075 | GSE14520 | 1.431510793 | 0.186563414 | 5.529442972 |
| GSM363149 | GSE14520 | 1.45318237 | 0.186671372 | 6.277646467 |
| GSM363432 | GSE14520 | 1.338476543 | 0.187112825 | 5.57114927 |
| GSM363174 | GSE14520 | 1.381878996 | 0.187184697 | 6.154537423 |
| GSM362960 | GSE14520 | 1.435039918 | 0.187230993 | 5.935967667 |
| GSM363172 | GSE14520 | 1.452464932 | 0.187275247 | 5.418183072 |
| GSM362958 | GSE14520 | 1.455988796 | 0.18760032 | 5.741428004 |
| GSM363207 | GSE14520 | 1.523757792 | 0.188200897 | 5.859271639 |
| GSM363204 | GSE14520 | 1.606666851 | 0.1882675 | 6.053811303 |
| GSM362959 | GSE14520 | 1.623067251 | 0.189176418 | 5.481383775 |
| GSM363442 | GSE14520 | 1.688588386 | 0.189397294 | 6.245371645 |
| GSM363031 | GSE14520 | 1.65621 | 0.189544461 | 6.265796335 |
| GSM363298 | GSE14520 | 1.620147386 | 0.189584183 | 4.904348521 |
| GSM363426 | GSE14520 | 1.401149738 | 0.190430428 | 4.537232501 |
| GSM363051 | GSE14520 | 1.955543398 | 0.191015463 | 5.79771613 |
| GSM363422 | GSE14520 | 1.720353029 | 0.191311723 | 6.198171574 |
| GSM363115 | GSE14520 | 1.518466565 | 0.191607113 | 5.307581842 |
| GSM363104 | GSE14520 | 1.10719065 | 0.191636729 | 4.894473411 |
| GSM363015 | GSE14520 | 1.396998535 | 0.191683596 | 6.21049572 |
| GSM363100 | GSE14520 | 1.509897007 | 0.191820041 | 5.504425369 |
| GSM363371 | GSE14520 | 1.604347783 | 0.192126069 | 5.669998372 |
| GSM363086 | GSE14520 | 1.439723272 | 0.192222131 | 5.411599336 |
| GSM363213 | GSE14520 | 1.19406926 | 0.192834956 | 7.417234139 |
| GSM363376 | GSE14520 | 1.662714426 | 0.193082852 | 6.058419029 |
| GSM363346 | GSE14520 | 1.514262803 | 0.193549064 | 5.49619644 |
| GSM362970 | GSE14520 | 1.57152716 | 0.193702334 | 5.702915076 |
| GSM363218 | GSE14520 | 1.770916089 | 0.193709064 | 6.387918855 |
| GSM363337 | GSE14520 | 1.505106811 | 0.194915065 | 4.709479029 |
| GSM363053 | GSE14520 | 1.577166042 | 0.195146025 | 5.72760285 |
| GSM363317 | GSE14520 | 1.56828907 | 0.195427932 | 5.955060205 |
| GSM363079 | GSE14520 | 1.420888192 | 0.195553808 | 5.049841476 |
| GSM363166 | GSE14520 | 1.581264366 | 0.195845948 | 6.52320786 |
| GSM363209 | GSE14520 | 1.598416405 | 0.196012625 | 5.23450603 |
| GSM363384 | GSE14520 | 1.869825966 | 0.196457605 | 6.125899604 |
| GSM363146 | GSE14520 | 1.612866318 | 0.196585328 | 5.412586847 |
| GSM363069 | GSE14520 | 1.533106659 | 0.197298875 | 6.333934593 |
| GSM363211 | GSE14520 | 1.5058806 | 0.197304313 | 5.424766808 |
| GSM363030 | GSE14520 | 1.567385347 | 0.198214673 | 5.693369795 |
| GSM363184 | GSE14520 | 1.723397397 | 0.198423632 | 6.771073116 |
| GSM363196 | GSE14520 | 1.31905715 | 0.199237919 | 5.4352996 |
| GSM363329 | GSE14520 | 1.44468611 | 0.199629124 | 5.353994858 |
| GSM363357 | GSE14520 | 1.472649914 | 0.199998779 | 5.658477082 |
| GSM363017 | GSE14520 | 1.663428617 | 0.200154737 | 6.587724915 |
| GSM712542 | GSE14520 | 1.560759352 | 0.200227396 | 5.721020102 |
| GSM363391 | GSE14520 | 1.397547787 | 0.200280875 | 5.554130746 |
| GSM363249 | GSE14520 | 1.481645076 | 0.200516491 | 6.71544366 |
| GSM363078 | GSE14520 | 1.539487815 | 0.201161498 | 5.576843499 |
| GSM363013 | GSE14520 | 1.664256252 | 0.202247314 | 5.473812528 |
| GSM363311 | GSE14520 | 1.5147426 | 0.202250866 | 6.250654829 |
| GSM363083 | GSE14520 | 1.711148601 | 0.202384777 | 5.64794429 |
| GSM363080 | GSE14520 | 1.693009009 | 0.202497554 | 5.997193348 |
| GSM363386 | GSE14520 | 1.807886353 | 0.20273432 | 5.47710489 |
| GSM363170 | GSE14520 | 1.561303304 | 0.203868149 | 6.236171005 |
| GSM363297 | GSE14520 | 1.865445404 | 0.203946685 | 6.101540671 |
| GSM363232 | GSE14520 | 1.63130431 | 0.205131797 | 5.876388167 |
| GSM363446 | GSE14520 | 1.297368717 | 0.205748975 | 5.888118612 |
| GSM363198 | GSE14520 | 1.41184126 | 0.206336796 | 5.046220274 |
| GSM363332 | GSE14520 | 1.660999441 | 0.206497645 | 6.744739161 |
| GSM363350 | GSE14520 | 1.730174622 | 0.207765664 | 5.590009983 |
| GSM363190 | GSE14520 | 1.669337528 | 0.207823814 | 6.253617362 |
| GSM362992 | GSE14520 | 1.434984522 | 0.208127857 | 5.906672167 |
| GSM363274 | GSE14520 | 1.454345951 | 0.208982456 | 5.508705241 |
| GSM363147 | GSE14520 | 1.644307306 | 0.209192156 | 5.302973129 |
| GSM363180 | GSE14520 | 1.417965364 | 0.210013524 | 6.608133804 |
| GSM363309 | GSE14520 | 1.709326233 | 0.210620305 | 5.743403026 |
| GSM363057 | GSE14520 | 1.222544085 | 0.210721952 | 6.074877874 |
| GSM363009 | GSE14520 | 1.700891141 | 0.211789845 | 5.951110161 |
| GSM363052 | GSE14520 | 1.702415896 | 0.212158775 | 6.198975417 |
| GSM363268 | GSE14520 | 1.60592077 | 0.213048588 | 6.13182467 |
| GSM363310 | GSE14520 | 1.636401677 | 0.213050354 | 5.995876996 |
| GSM363296 | GSE14520 | 1.503868892 | 0.213140825 | 5.741757833 |
| GSM363105 | GSE14520 | 1.45469287 | 0.21322857 | 7.365883568 |
| GSM363077 | GSE14520 | 1.360229434 | 0.213781363 | 6.633480248 |
| GSM363049 | GSE14520 | 1.515859177 | 0.214508052 | 6.109111918 |
| GSM363038 | GSE14520 | 1.523373946 | 0.214638836 | 6.004105925 |
| GSM363341 | GSE14520 | 1.482975382 | 0.216354065 | 5.823721244 |
| GSM363348 | GSE14520 | 1.6675523 | 0.216568828 | 5.58770612 |
| GSM363226 | GSE14520 | 1.123806614 | 0.217258724 | 5.399091522 |
| GSM363235 | GSE14520 | 1.778014166 | 0.217424086 | 6.468565915 |
| GSM363271 | GSE14520 | 1.76992428 | 0.218497216 | 6.34183468 |
| GSM363420 | GSE14520 | 1.746725709 | 0.220173662 | 6.474859664 |
| GSM363263 | GSE14520 | 1.743482493 | 0.221426398 | 6.618667584 |
| GSM362983 | GSE14520 | 1.350703954 | 0.221454677 | 5.919509809 |
| GSM363330 | GSE14520 | 1.654567385 | 0.223858327 | 6.161120171 |
| GSM362986 | GSE14520 | 1.72524519 | 0.22446676 | 5.484017467 |
| GSM363404 | GSE14520 | 1.155522045 | 0.226284762 | 6.031427391 |
| GSM363237 | GSE14520 | 1.94228999 | 0.22686899 | 6.141369951 |
| GSM363032 | GSE14520 | 1.800072313 | 0.227347275 | 6.008055969 |
| GSM362956 | GSE14520 | 1.625285752 | 0.229230044 | 6.462652855 |
| GSM363150 | GSE14520 | 1.65549724 | 0.235126862 | 5.74735307 |
| GSM363085 | GSE14520 | 1.703955551 | 0.235162886 | 5.832937684 |
| GSM363194 | GSE14520 | 1.869575598 | 0.236908795 | 6.190087818 |
| GSM363129 | GSE14520 | 1.863370013 | 0.236989494 | 6.641050508 |
| GSM363245 | GSE14520 | 2.049329063 | 0.241471391 | 6.403060361 |
| GSM363070 | GSE14520 | 1.654583132 | 0.249205846 | 7.895848123 |
| GSM363222 | GSE14520 | 1.712559562 | 0.25138132 | 7.034738548 |
| GSM363354 | GSE14520 | 1.724534811 | 0.27075615 | 5.876388167 |
| TCGA-2Y-A9H2-01A-12R-A38B-07 | LIHC | 3.231747041 | 0.575219437 | 2.468545487 |
| TCGA-DD-A73C-01A-12R-A33J-07 | LIHC | 3.431510288 | 0.588574611 | 1.893057561 |
| TCGA-DD-A3A6-01A-11R-A22L-07 | LIHC | 3.284655213 | 0.589854285 | 0.508505838 |
| TCGA-DD-A3A2-01A-11R-A213-07 | LIHC | 3.261321281 | 0.59279681 | 0.714202469 |
| TCGA-ED-A82E-01A-11R-A352-07 | LIHC | 3.315714444 | 0.595994222 | 1.975516235 |
| TCGA-2Y-A9H6-01A-11R-A39D-07 | LIHC | 3.294379837 | 0.596420969 | 2.859093586 |
| TCGA-2Y-A9GZ-01A-11R-A39D-07 | LIHC | 3.240898614 | 0.596556674 | 2.042760038 |
| TCGA-ES-A2HS-01A-11R-A180-07 | LIHC | 3.346143848 | 0.597839599 | 1.553150591 |
| TCGA-2Y-A9H3-01A-11R-A38B-07 | LIHC | 3.368177682 | 0.600354603 | 1.497729373 |
| TCGA-FV-A3I0-01A-11R-A22L-07 | LIHC | 3.367692371 | 0.602479845 | 1.57308965 |
| TCGA-NI-A8LF-01A-11R-A36F-07 | LIHC | 3.219829279 | 0.604456255 | 0.936521543 |
| TCGA-CC-A7IF-01A-11R-A33J-07 | LIHC | 3.235073259 | 0.605414693 | 2.703061576 |
| TCGA-BC-A216-01A-11R-A155-07 | LIHC | 3.406984289 | 0.605812458 | 3.502726203 |
| TCGA-G3-A6UC-01A-21R-A33J-07 | LIHC | 3.04837574 | 0.605932355 | 3.231498035 |
| TCGA-DD-AAEB-01A-11R-A41C-07 | LIHC | 3.200720178 | 0.606825978 | 0.72185592 |
| TCGA-LG-A6GG-01A-11R-A311-07 | LIHC | 3.417831663 | 0.60687655 | 3.40553927 |
| TCGA-DD-AADA-01A-11R-A41C-07 | LIHC | 3.367481132 | 0.607344683 | 1.396504268 |
| TCGA-UB-A7ME-01A-11R-A33J-07 | LIHC | 3.381497736 | 0.607499647 | 3.248554298 |
| TCGA-DD-A4ND-01A-11R-A266-07 | LIHC | 3.451343478 | 0.607659582 | 3.079160799 |
| TCGA-KR-A7K2-01A-12R-A33R-07 | LIHC | 3.241409331 | 0.60923289 | 1.829911966 |
| TCGA-DD-A4NS-01A-11R-A311-07 | LIHC | 3.263981498 | 0.609766394 | 1.449464915 |
| TCGA-GJ-A6C0-01A-12R-A311-07 | LIHC | 3.492379416 | 0.610214871 | 3.315622685 |
| TCGA-DD-A4NJ-01A-11R-A27V-07 | LIHC | 3.460617878 | 0.61184142 | 3.901676606 |
| TCGA-DD-AAW1-01A-11R-A41C-07 | LIHC | 3.158343486 | 0.611924567 | 2.028085649 |
| TCGA-DD-AAEA-01A-11R-A41C-07 | LIHC | 3.396419925 | 0.612257064 | 3.746601515 |
| TCGA-ED-A5KG-01A-11R-A27V-07 | LIHC | 3.611419334 | 0.612689204 | 3.178065047 |
| TCGA-FV-A4ZP-01A-12R-A266-07 | LIHC | 3.230312424 | 0.613230001 | 2.3609151 |
| TCGA-ZS-A9CF-02A-11R-A38B-07 | LIHC | 3.337369382 | 0.6134639 | 3.315536842 |
| TCGA-DD-A73G-01A-22R-A32O-07 | LIHC | 3.234965946 | 0.614779573 | 3.1207581 |
| TCGA-DD-A3A9-01A-11R-A266-07 | LIHC | 3.471514045 | 0.614862043 | 1.864536862 |
| TCGA-BC-A10Y-01A-11R-A131-07 | LIHC | 3.427474229 | 0.615069276 | 4.238932822 |
| TCGA-G3-A5SK-01A-11R-A27V-07 | LIHC | 3.208129533 | 0.615534864 | 3.522760636 |
| TCGA-ED-A7PY-01A-11R-A33R-07 | LIHC | 3.328568941 | 0.616007011 | 2.954508555 |
| TCGA-DD-AAE2-01A-11R-A41C-07 | LIHC | 3.385776076 | 0.616360105 | 2.371574672 |
| TCGA-BC-4072-01B-11R-A155-07 | LIHC | 3.512616556 | 0.616448122 | 4.516730394 |
| TCGA-2Y-A9GT-01A-11R-A38B-07 | LIHC | 3.302472305 | 0.616596308 | 2.251075445 |
| TCGA-ED-A8O5-01A-11R-A36F-07 | LIHC | 3.511945438 | 0.616821487 | 2.202573027 |
| TCGA-4R-AA8I-01A-11R-A38B-07 | LIHC | 3.275499685 | 0.616993754 | 3.275840067 |
| TCGA-ZS-A9CF-01A-11R-A38B-07 | LIHC | 3.343388696 | 0.617511223 | 3.304089379 |
| TCGA-ZP-A9D2-01A-11R-A38B-07 | LIHC | 3.524118328 | 0.618684924 | 3.38082067 |
| TCGA-2Y-A9H5-01A-11R-A38B-07 | LIHC | 3.435679273 | 0.618805862 | 2.385477253 |
| TCGA-ZP-A9D4-01A-11R-A37K-07 | LIHC | 3.131090589 | 0.620134412 | 1.700853537 |
| TCGA-DD-AAVQ-01A-11R-A41C-07 | LIHC | 3.523136125 | 0.620764489 | 2.775655604 |
| TCGA-DD-A3A7-01A-11R-A22L-07 | LIHC | 3.268791183 | 0.620806219 | 2.742988738 |
| TCGA-CC-A7IH-01A-11R-A33J-07 | LIHC | 3.221807107 | 0.621045071 | 2.82590765 |
| TCGA-G3-AAUZ-01A-11R-A38B-07 | LIHC | 3.470164166 | 0.621269664 | 3.355310793 |
| TCGA-DD-A1EB-01A-11R-A131-07 | LIHC | 3.40361833 | 0.621343991 | 1.812399265 |
| TCGA-G3-A7M6-01A-11R-A33R-07 | LIHC | 3.299479034 | 0.621578995 | 3.271730771 |
| TCGA-2Y-A9H9-01A-21R-A39D-07 | LIHC | 3.331109527 | 0.621761848 | 4.280109368 |
| TCGA-DD-A4NK-01A-11R-A28V-07 | LIHC | 3.201815328 | 0.621877782 | 1.371715972 |
| TCGA-G3-A25T-01A-11R-A16W-07 | LIHC | 3.41807968 | 0.621935392 | 2.681424685 |
| TCGA-HP-A5N0-01A-11R-A28V-07 | LIHC | 3.292075933 | 0.62199139 | 1.082761594 |
| TCGA-WQ-A9G7-01A-11R-A37K-07 | LIHC | 3.283172383 | 0.621991996 | 3.396222248 |
| TCGA-G3-A25S-01A-11R-A16W-07 | LIHC | 3.397574605 | 0.622884808 | 3.484358001 |
| TCGA-DD-AAVW-01A-11R-A41C-07 | LIHC | 3.446625172 | 0.623439049 | 2.318893737 |
| TCGA-DD-AADF-01A-11R-A41C-07 | LIHC | 3.39201036 | 0.624177509 | 3.983383358 |
| TCGA-DD-A4NI-01A-11R-A27V-07 | LIHC | 3.276660979 | 0.624241026 | 1.351573785 |
| TCGA-BC-A217-01A-11R-A155-07 | LIHC | 3.379010104 | 0.624963153 | 4.393930195 |
| TCGA-DD-AACO-01A-11R-A41C-07 | LIHC | 3.437695486 | 0.625094882 | 2.581784745 |
| TCGA-DD-AACQ-01A-11R-A41C-07 | LIHC | 3.192771842 | 0.62561395 | 1.917151487 |
| TCGA-RC-A7SH-01A-11R-A38B-07 | LIHC | 3.28415352 | 0.626172923 | 2.52207821 |
| TCGA-DD-AADW-01A-11R-A39D-07 | LIHC | 3.547170161 | 0.626217064 | 4.487044239 |
| TCGA-DD-AAD8-01A-11R-A41C-07 | LIHC | 3.229463257 | 0.626338246 | 2.196760841 |
| TCGA-G3-AAV0-01A-11R-A37K-07 | LIHC | 3.263780623 | 0.626426874 | 1.032706601 |
| TCGA-FV-A495-01A-11R-A266-07 | LIHC | 3.453939445 | 0.626466564 | 2.63489381 |
| TCGA-DD-AACN-01A-11R-A41C-07 | LIHC | 3.385206432 | 0.626801152 | 2.367027142 |
| TCGA-RC-A7SK-01A-11R-A352-07 | LIHC | 3.262879141 | 0.626863613 | 2.641753316 |
| TCGA-CC-5264-01A-01R-A131-07 | LIHC | 3.636858564 | 0.626979838 | 5.008323382 |
| TCGA-2Y-A9GU-01A-11R-A38B-07 | LIHC | 3.465997691 | 0.627352191 | 3.864231387 |
| TCGA-DD-AAEH-01A-11R-A41C-07 | LIHC | 3.446134942 | 0.627537623 | 2.591899103 |
| TCGA-DD-AADS-01A-11R-A41C-07 | LIHC | 3.171348623 | 0.627666602 | 1.730715001 |
| TCGA-CC-A9FS-01A-11R-A37K-07 | LIHC | 3.410525919 | 0.628112745 | 4.632429504 |
| TCGA-WJ-A86L-01A-12R-A39D-07 | LIHC | 3.034230499 | 0.628145393 | 1.862402843 |
| TCGA-BD-A2L6-01A-11R-A213-07 | LIHC | 3.399051643 | 0.628222656 | 2.834555867 |
| TCGA-RC-A7SF-01A-11R-A352-07 | LIHC | 3.278254279 | 0.628255241 | 1.720940645 |
| TCGA-MI-A75H-01A-11R-A32O-07 | LIHC | 3.355209118 | 0.628286109 | 3.032292949 |
| TCGA-CC-A3MA-01A-11R-A213-07 | LIHC | 3.604006217 | 0.628341873 | 3.29017244 |
| TCGA-EP-A2KC-01A-11R-A213-07 | LIHC | 3.297651614 | 0.628513614 | 2.19608561 |
| TCGA-FV-A3R3-01A-11R-A22L-07 | LIHC | 3.521734799 | 0.628698161 | 2.457305844 |
| TCGA-UB-A7MA-01A-11R-A33R-07 | LIHC | 3.499376696 | 0.628895483 | 4.015605856 |
| TCGA-DD-A4NV-01A-11R-A311-07 | LIHC | 3.247723799 | 0.628957176 | 2.783888175 |
| TCGA-DD-AA3A-01A-11R-A37K-07 | LIHC | 3.293484731 | 0.629301332 | 3.582000638 |
| TCGA-2Y-A9GV-01A-11R-A38B-07 | LIHC | 3.264178942 | 0.629564282 | 1.050295066 |
| TCGA-XR-A8TG-01A-11R-A36F-07 | LIHC | 3.479549821 | 0.629669551 | 4.027123397 |
| TCGA-KR-A7K8-01A-11R-A33J-07 | LIHC | 3.303392205 | 0.630060385 | 2.200042933 |
| TCGA-CC-5261-01A-01R-A131-07 | LIHC | 3.593360556 | 0.630119847 | 4.558141509 |
| TCGA-DD-AAE3-01A-11R-A41C-07 | LIHC | 3.398023101 | 0.630764168 | 1.829658103 |
| TCGA-G3-AAV2-01A-11R-A37K-07 | LIHC | 3.23330439 | 0.630893964 | 2.032883009 |
| TCGA-DD-A73E-01A-12R-A32O-07 | LIHC | 3.270278109 | 0.631006164 | 2.794146422 |
| TCGA-DD-AADG-01A-11R-A41C-07 | LIHC | 3.320494641 | 0.631238652 | 2.343316399 |
| TCGA-MI-A75G-01A-11R-A32O-07 | LIHC | 3.215966374 | 0.631533882 | 3.766251373 |
| TCGA-ZS-A9CE-01A-11R-A37K-07 | LIHC | 3.120345631 | 0.63157223 | 1.850096575 |
| TCGA-WX-AA44-01A-11R-A39D-07 | LIHC | 3.451537177 | 0.63165115 | 2.686620977 |
| TCGA-MR-A8JO-01A-12R-A36F-07 | LIHC | 3.370956908 | 0.631713749 | 1.96204537 |
| TCGA-BC-A10Q-01A-11R-A131-07 | LIHC | 3.41881973 | 0.631917113 | 3.74410455 |
| TCGA-DD-AADY-01A-11R-A41C-07 | LIHC | 3.58026043 | 0.63197422 | 5.312267747 |
| TCGA-DD-A1ED-01A-11R-A155-07 | LIHC | 3.270912844 | 0.632280238 | 0.995818986 |
| TCGA-DD-A4NL-01A-11R-A28V-07 | LIHC | 3.213874582 | 0.632711639 | 0.881699662 |
| TCGA-LG-A9QC-01A-11R-A37K-07 | LIHC | 3.343811302 | 0.63279939 | 1.993114002 |
| TCGA-DD-AACA-02A-11R-A41C-07 | LIHC | 3.267992149 | 0.633010593 | 2.518496398 |
| TCGA-YA-A8S7-01A-11R-A37K-07 | LIHC | 3.540168821 | 0.63302319 | 3.955772784 |
| TCGA-DD-AADV-01A-11R-A39D-07 | LIHC | 3.466002821 | 0.63303811 | 3.448772321 |
| TCGA-BC-A10U-01A-11R-A131-07 | LIHC | 3.206487631 | 0.633379834 | 2.11004046 |
| TCGA-ED-A627-01A-12R-A311-07 | LIHC | 3.519240435 | 0.63347161 | 1.650445598 |
| TCGA-ZS-A9CG-01A-11R-A37K-07 | LIHC | 3.18716472 | 0.634760405 | 1.177099461 |
| TCGA-DD-A4NN-01A-11R-A28V-07 | LIHC | 3.488410252 | 0.634859076 | 4.17585574 |
| TCGA-DD-A1EH-01A-11R-A131-07 | LIHC | 3.59770159 | 0.634874609 | 3.386128736 |
| TCGA-CC-A8HS-01A-11R-A36F-07 | LIHC | 3.546113754 | 0.635213678 | 4.571556867 |
| TCGA-RC-A6M5-01A-11R-A32O-07 | LIHC | 3.27496526 | 0.635414741 | 1.998523271 |
| TCGA-ED-A97K-01A-21R-A38B-07 | LIHC | 3.268286722 | 0.635524472 | 2.725787001 |
| TCGA-G3-A5SJ-01A-11R-A27V-07 | LIHC | 3.411461803 | 0.635611894 | 2.388182079 |
| TCGA-KR-A7K0-01A-12R-A33R-07 | LIHC | 3.260361448 | 0.635614112 | 2.129886926 |
| TCGA-ED-A7XP-01A-11R-A352-07 | LIHC | 3.421567226 | 0.635720213 | 2.443988849 |
| TCGA-DD-AACI-01A-11R-A41C-07 | LIHC | 3.445814976 | 0.635850186 | 2.041571517 |
| TCGA-2Y-A9H8-01A-11R-A39D-07 | LIHC | 3.383956918 | 0.635942596 | 2.89820195 |
| TCGA-DD-A4NA-01A-11R-A266-07 | LIHC | 3.396869417 | 0.635949357 | 3.565378328 |
| TCGA-DD-AAVP-01A-11R-A41C-07 | LIHC | 3.432169522 | 0.636231541 | 4.005046888 |
| TCGA-DD-AADO-01A-11R-A41C-07 | LIHC | 3.382859087 | 0.636472391 | 2.018416468 |
| TCGA-K7-AAU7-01A-11R-A38B-07 | LIHC | 3.425696753 | 0.636948151 | 3.222880388 |
| TCGA-DD-AAD3-01A-11R-A41C-07 | LIHC | 3.558508115 | 0.637110006 | 2.743796031 |
| TCGA-ED-A4XI-01A-11R-A266-07 | LIHC | 3.397343757 | 0.637146463 | 1.506800941 |
| TCGA-MI-A75E-01A-11R-A32O-07 | LIHC | 3.410368148 | 0.637564014 | 2.245712891 |
| TCGA-K7-A5RG-01A-11R-A28V-07 | LIHC | 3.544402332 | 0.63767523 | 2.749637404 |
| TCGA-DD-AAED-01A-12R-A41C-07 | LIHC | 3.21504115 | 0.637878583 | 1.3747017 |
| TCGA-XR-A8TC-01A-11R-A36F-07 | LIHC | 3.331322818 | 0.6379078 | 4.682675912 |
| TCGA-DD-A11A-01A-11R-A131-07 | LIHC | 3.345813849 | 0.638252118 | 3.767929886 |
| TCGA-DD-AAW3-01A-11R-A41C-07 | LIHC | 3.273996095 | 0.638340969 | 1.803220827 |
| TCGA-BC-A3KF-01A-11R-A213-07 | LIHC | 3.284010346 | 0.638526388 | 2.86970087 |
| TCGA-DD-AAEE-01A-11R-A41C-07 | LIHC | 3.404771291 | 0.638565663 | 2.590408002 |
| TCGA-DD-A116-01A-11R-A131-07 | LIHC | 3.368222889 | 0.638621098 | 2.468734947 |
| TCGA-5C-A9VH-01A-11R-A37K-07 | LIHC | 3.381659421 | 0.638718289 | 2.32696708 |
| TCGA-ED-A7PX-01A-51R-A352-07 | LIHC | 3.553221233 | 0.638725723 | 3.404913786 |
| TCGA-ZP-A9D1-01A-11R-A38B-07 | LIHC | 3.491453321 | 0.638935714 | 2.620977201 |
| TCGA-RC-A7S9-01A-11R-A33R-07 | LIHC | 3.432281701 | 0.638949876 | 3.594615956 |
| TCGA-DD-A3A5-01A-11R-A22L-07 | LIHC | 3.410091767 | 0.639306728 | 2.790433715 |
| TCGA-DD-A4NH-01A-11R-A27V-07 | LIHC | 3.381688659 | 0.639333448 | 3.282410685 |
| TCGA-DD-AADU-01A-11R-A41C-07 | LIHC | 3.16114773 | 0.639654911 | 1.531766269 |
| TCGA-DD-AAVS-01A-11R-A41C-07 | LIHC | 3.492465268 | 0.640622613 | 3.153124609 |
| TCGA-5C-AAPD-01A-21R-A39D-07 | LIHC | 3.536172045 | 0.640747002 | 3.145935001 |
| TCGA-WQ-AB4B-01A-11R-A41C-07 | LIHC | 3.455177909 | 0.641050889 | 2.281606878 |
| TCGA-NI-A4U2-01A-11R-A28V-07 | LIHC | 3.293004062 | 0.641231543 | 1.891209026 |
| TCGA-DD-AACT-01A-11R-A41C-07 | LIHC | 3.416946319 | 0.641577268 | 1.795603779 |
| TCGA-DD-AAE0-01A-11R-A41C-07 | LIHC | 3.550908142 | 0.64173642 | 4.595404115 |
| TCGA-2Y-A9H4-01A-11R-A38B-07 | LIHC | 3.393151166 | 0.641764443 | 2.107527991 |
| TCGA-G3-A3CI-01A-11R-A213-07 | LIHC | 3.26773856 | 0.641930194 | 0.619590718 |
| TCGA-DD-A4NR-01A-11R-A311-07 | LIHC | 3.55842569 | 0.642022549 | 4.24425136 |
| TCGA-CC-A8HV-01A-11R-A36F-07 | LIHC | 3.27215351 | 0.642327034 | 2.61149887 |
| TCGA-EP-A2KB-01A-11R-A180-07 | LIHC | 3.505902123 | 0.642439736 | 3.579228528 |
| TCGA-DD-AAE4-01A-11R-A41C-07 | LIHC | 3.534422425 | 0.642575227 | 2.294277291 |
| TCGA-DD-AACJ-01A-11R-A41C-07 | LIHC | 3.173148705 | 0.642597262 | 1.314083408 |
| TCGA-2Y-A9HB-01A-11R-A39D-07 | LIHC | 3.401837707 | 0.642597269 | 2.494775454 |
| TCGA-UB-A7MC-01A-11R-A33R-07 | LIHC | 3.302352395 | 0.642988633 | 2.912837924 |
| TCGA-DD-A11C-01A-11R-A131-07 | LIHC | 3.619508607 | 0.643092896 | 4.756363872 |
| TCGA-DD-AAE1-01A-11R-A41C-07 | LIHC | 3.485951985 | 0.643320491 | 1.607705251 |
| TCGA-EP-A2KA-01A-11R-A180-07 | LIHC | 3.49447705 | 0.64335575 | 3.38877426 |
| TCGA-DD-A4NF-01A-11R-A27V-07 | LIHC | 3.201523419 | 0.643486199 | 2.552101694 |
| TCGA-WX-AA46-01A-11R-A39D-07 | LIHC | 3.25339586 | 0.643488435 | 0.656012969 |
| TCGA-DD-AACF-01A-11R-A41C-07 | LIHC | 3.561028432 | 0.643661685 | 4.588984224 |
| TCGA-DD-AAD1-01A-11R-A41C-07 | LIHC | 3.505163121 | 0.643728529 | 2.136836751 |
| TCGA-2Y-A9GX-01A-11R-A38B-07 | LIHC | 3.482853155 | 0.643810333 | 2.492169772 |
| TCGA-ED-A66Y-01A-11R-A311-07 | LIHC | 3.36182836 | 0.644015929 | 2.81892483 |
| TCGA-HP-A5MZ-01A-21R-A27V-07 | LIHC | 3.404622692 | 0.644163068 | 2.181114432 |
| TCGA-BC-A110-01A-11R-A131-07 | LIHC | 3.406160261 | 0.644205612 | 0.816734373 |
| TCGA-BC-A69I-01A-11R-A311-07 | LIHC | 3.334306479 | 0.644240786 | 2.571596444 |
| TCGA-DD-AAVV-01A-11R-A41C-07 | LIHC | 3.501854318 | 0.644537486 | 3.463444464 |
| TCGA-DD-AACC-01A-11R-A41C-07 | LIHC | 3.537579313 | 0.644725338 | 4.261710166 |
| TCGA-DD-A4NE-01A-11R-A27V-07 | LIHC | 3.473776638 | 0.644735761 | 4.394922031 |
| TCGA-CC-5260-01A-01R-A131-07 | LIHC | 3.680353978 | 0.64476171 | 3.554319162 |
| TCGA-2Y-A9GW-01A-11R-A38B-07 | LIHC | 3.44263031 | 0.644816622 | 1.874635914 |
| TCGA-5R-AAAM-01A-12R-A41C-07 | LIHC | 3.477808162 | 0.644861356 | 1.591349929 |
| TCGA-DD-A113-01A-11R-A131-07 | LIHC | 3.534621828 | 0.645002203 | 3.255308168 |
| TCGA-DD-AAVR-01A-11R-A41C-07 | LIHC | 3.5010343 | 0.64524974 | 2.531011423 |
| TCGA-DD-AADK-01A-11R-A41C-07 | LIHC | 3.356200926 | 0.645350619 | 1.850182198 |
| TCGA-DD-A3A1-01A-11R-A213-07 | LIHC | 3.252494927 | 0.64539414 | 1.846405631 |
| TCGA-DD-A4NP-01A-11R-A28V-07 | LIHC | 3.051359594 | 0.645604052 | 1.051236769 |
| TCGA-FV-A23B-01A-11R-A16W-07 | LIHC | 3.6170449 | 0.645708221 | 4.06547631 |
| TCGA-G3-AAV6-01A-21R-A37K-07 | LIHC | 3.480398135 | 0.645825897 | 4.307576987 |
| TCGA-K7-A5RF-01A-11R-A28V-07 | LIHC | 3.45295864 | 0.645913797 | 1.55189214 |
| TCGA-UB-AA0U-01A-11R-A38B-07 | LIHC | 3.541892447 | 0.646031522 | 3.751883086 |
| TCGA-CC-A7IL-01A-11R-A33R-07 | LIHC | 3.300030734 | 0.646165022 | 2.884012324 |
| TCGA-DD-A11B-01A-11R-A131-07 | LIHC | 3.434478006 | 0.646260182 | 3.794438827 |
| TCGA-EP-A3JL-01A-11R-A213-07 | LIHC | 3.50805877 | 0.646362868 | 2.352484295 |
| TCGA-DD-AADC-01A-11R-A41C-07 | LIHC | 3.577705837 | 0.646512629 | 3.826155585 |
| TCGA-2Y-A9H1-01A-11R-A38B-07 | LIHC | 3.252106013 | 0.646644171 | 0.828674605 |
| TCGA-DD-AAE7-01A-11R-A41C-07 | LIHC | 3.396356593 | 0.646840513 | 1.154522582 |
| TCGA-GJ-A3OU-01A-31R-A38B-07 | LIHC | 3.592681925 | 0.647160184 | 3.061580677 |
| TCGA-UB-A7MB-01A-11R-A33R-07 | LIHC | 3.356687141 | 0.647244069 | 4.022415732 |
| TCGA-DD-A73D-01A-12R-A32O-07 | LIHC | 3.170926838 | 0.647289652 | 2.006109643 |
| TCGA-EP-A12J-01A-11R-A131-07 | LIHC | 3.374101467 | 0.647330374 | 2.358434968 |
| TCGA-DD-AAD5-01A-11R-A41C-07 | LIHC | 3.491145217 | 0.647331747 | 3.75338771 |
| TCGA-DD-AACE-01A-11R-A41C-07 | LIHC | 3.40028908 | 0.647349457 | 1.545528764 |
| TCGA-DD-AADI-01A-11R-A41C-07 | LIHC | 3.429034006 | 0.647469636 | 2.22056193 |
| TCGA-DD-A3A8-01A-11R-A22L-07 | LIHC | 3.282208007 | 0.647492314 | 1.652042265 |
| TCGA-3K-AAZ8-01A-12R-A39D-07 | LIHC | 3.283651336 | 0.647555216 | 2.248288572 |
| TCGA-WX-AA47-01A-11R-A39D-07 | LIHC | 3.279692483 | 0.647878099 | 1.931490191 |
| TCGA-FV-A496-01A-11R-A266-07 | LIHC | 3.176781565 | 0.648064305 | 1.965660695 |
| TCGA-ED-A7XO-01A-11R-A352-07 | LIHC | 3.466943515 | 0.64807018 | 3.322556375 |
| TCGA-DD-AACA-01A-11R-A41C-07 | LIHC | 3.491266386 | 0.648197198 | 3.284447866 |
| TCGA-DD-A73A-01A-12R-A32O-07 | LIHC | 3.383636166 | 0.648398061 | 1.608922836 |
| TCGA-DD-AADQ-01A-11R-A41C-07 | LIHC | 3.366475503 | 0.648492745 | 2.193227777 |
| TCGA-G3-AAV3-01A-11R-A37K-07 | LIHC | 3.463406722 | 0.648500945 | 2.369624737 |
| TCGA-G3-A7M5-01A-11R-A33R-07 | LIHC | 3.212043264 | 0.6486391 | 3.489639876 |
| TCGA-DD-AACS-01A-11R-A41C-07 | LIHC | 3.493553014 | 0.648668271 | 6.659069507 |
| TCGA-DD-A1EA-01A-11R-A131-07 | LIHC | 3.458133283 | 0.648913492 | 2.412643057 |
| TCGA-DD-AAE6-01A-11R-A41C-07 | LIHC | 3.267780263 | 0.648985664 | 4.354181854 |
| TCGA-DD-AAW2-01A-11R-A41C-07 | LIHC | 3.319419116 | 0.649295385 | 1.542015078 |
| TCGA-BC-A10R-01A-11R-A131-07 | LIHC | 3.485075771 | 0.649316944 | 1.122429546 |
| TCGA-DD-A115-01A-11R-A131-07 | LIHC | 3.578678026 | 0.649324762 | 3.556238406 |
| TCGA-ZP-A9CZ-01A-11R-A38B-07 | LIHC | 3.654635788 | 0.649611257 | 4.390983779 |
| TCGA-RC-A6M6-01A-11R-A32O-07 | LIHC | 3.546336524 | 0.6496213 | 4.693578176 |
| TCGA-BC-A5W4-01A-11R-A28V-07 | LIHC | 3.287676376 | 0.64977289 | 2.261056899 |
| TCGA-ED-A8O6-01A-11R-A36F-07 | LIHC | 3.379655279 | 0.649794314 | 4.026208832 |
| TCGA-DD-AACB-01A-11R-A41C-07 | LIHC | 3.587711711 | 0.64993978 | 3.01228898 |
| TCGA-FV-A2QR-01A-11R-A213-07 | LIHC | 3.461757512 | 0.650008966 | 2.484141672 |
| TCGA-ZP-A9D0-01A-11R-A37K-07 | LIHC | 3.21994168 | 0.650043837 | 5.6837016 |
| TCGA-EP-A3RK-01A-11R-A22L-07 | LIHC | 3.589969235 | 0.650402653 | 3.446452539 |
| TCGA-O8-A75V-01A-11R-A32O-07 | LIHC | 3.466621932 | 0.650487804 | 2.826932741 |
| TCGA-DD-AACY-01A-11R-A41C-07 | LIHC | 3.407759964 | 0.650514766 | 2.247548 |
| TCGA-DD-A1EC-01A-21R-A131-07 | LIHC | 3.582091084 | 0.650531226 | 3.276015286 |
| TCGA-DD-AACU-01A-11R-A41C-07 | LIHC | 3.509025004 | 0.650579646 | 2.527718196 |
| TCGA-MR-A520-01A-11R-A266-07 | LIHC | 3.32538301 | 0.650797812 | 1.16816261 |
| TCGA-DD-A4NG-01A-11R-A27V-07 | LIHC | 3.368191686 | 0.651051914 | 1.663265147 |
| TCGA-DD-AAC9-01A-11R-A41C-07 | LIHC | 3.474427113 | 0.651460821 | 1.098661852 |
| TCGA-BC-A10X-01A-11R-A131-07 | LIHC | 3.472468625 | 0.651482722 | 1.394164963 |
| TCGA-ZS-A9CD-01A-11R-A37K-07 | LIHC | 3.332939201 | 0.651545141 | 1.80543286 |
| TCGA-G3-A3CJ-01A-11R-A213-07 | LIHC | 3.318533683 | 0.651677965 | 1.372004518 |
| TCGA-ZP-A9CY-01A-11R-A38B-07 | LIHC | 3.50561917 | 0.65173158 | 1.788918914 |
| TCGA-DD-AACW-01A-11R-A41C-07 | LIHC | 3.548384191 | 0.651994924 | 3.214968411 |
| TCGA-UB-AA0V-01A-11R-A38B-07 | LIHC | 3.383884838 | 0.652073774 | 0.733347997 |
| TCGA-DD-A11D-01A-11R-A131-07 | LIHC | 3.446925997 | 0.652998957 | 2.608531866 |
| TCGA-BD-A3EP-01A-11R-A22L-07 | LIHC | 3.407784267 | 0.653190667 | 2.206875271 |
| TCGA-G3-A25Z-01A-11R-A16W-07 | LIHC | 3.505640026 | 0.653265997 | 3.354573214 |
| TCGA-DD-A119-01A-11R-A131-07 | LIHC | 3.462973161 | 0.65329363 | 1.970567046 |
| TCGA-XR-A8TF-01A-11R-A36F-07 | LIHC | 3.4582624 | 0.653320122 | 2.804470348 |
| TCGA-CC-A5UD-01A-11R-A28V-07 | LIHC | 3.556439834 | 0.653561731 | 4.520357572 |
| TCGA-ES-A2HT-01A-12R-A180-07 | LIHC | 3.377493336 | 0.653816006 | 2.486380079 |
| TCGA-DD-AADP-01A-11R-A39D-07 | LIHC | 3.474497755 | 0.653957043 | 3.883434756 |
| TCGA-DD-AAW0-01A-11R-A41C-07 | LIHC | 3.439433841 | 0.654100033 | 3.240538109 |
| TCGA-G3-A3CG-01A-11R-A213-07 | LIHC | 3.464129857 | 0.654146097 | 3.76457255 |
| TCGA-G3-A25V-01A-11R-A16W-07 | LIHC | 3.365832607 | 0.654161535 | 1.720684246 |
| TCGA-DD-A4NB-01A-12R-A266-07 | LIHC | 3.5055934 | 0.654407672 | 2.097349911 |
| TCGA-DD-A3A3-01A-11R-A22L-07 | LIHC | 3.310239054 | 0.654730879 | 2.193409563 |
| TCGA-CC-A3MB-01A-11R-A213-07 | LIHC | 3.543530508 | 0.654790464 | 4.170465612 |
| TCGA-ED-A66X-01A-11R-A311-07 | LIHC | 3.58559468 | 0.654930988 | 3.242684135 |
| TCGA-DD-AACG-01A-11R-A41C-07 | LIHC | 3.490491746 | 0.654939859 | 3.053466762 |
| TCGA-CC-A5UE-01A-11R-A28V-07 | LIHC | 3.470402423 | 0.655041623 | 4.393448013 |
| TCGA-T1-A6J8-01A-11R-A32O-07 | LIHC | 3.477898618 | 0.655290871 | 2.183382209 |
| TCGA-BC-A10S-01A-22R-A131-07 | LIHC | 3.445794912 | 0.655564815 | 1.102179382 |
| TCGA-CC-A7IG-01A-11R-A33J-07 | LIHC | 3.4602711 | 0.655684029 | 3.50930864 |
| TCGA-G3-A3CH-01A-11R-A22L-07 | LIHC | 3.494996937 | 0.655893009 | 2.380867292 |
| TCGA-DD-AAEK-01A-11R-A41C-07 | LIHC | 3.512728201 | 0.655936949 | 2.399334623 |
| TCGA-G3-AAV1-01A-11R-A38B-07 | LIHC | 3.45585968 | 0.656237973 | 2.615087675 |
| TCGA-G3-A5SL-01A-11R-A27V-07 | LIHC | 3.395228945 | 0.656440944 | 2.474645052 |
| TCGA-FV-A3R2-01A-11R-A22L-07 | LIHC | 3.420776428 | 0.656825618 | 3.800413337 |
| TCGA-MI-A75C-01A-11R-A32O-07 | LIHC | 3.226853422 | 0.657300421 | 1.878397121 |
| TCGA-DD-A4NO-01A-11R-A28V-07 | LIHC | 3.345188772 | 0.657599168 | 3.571892988 |
| TCGA-EP-A26S-01A-11R-A16W-07 | LIHC | 3.273964779 | 0.657906668 | 1.051380139 |
| TCGA-DD-A1EF-01A-11R-A131-07 | LIHC | 3.551831825 | 0.658314105 | 3.208579362 |
| TCGA-K7-A6G5-01A-11R-A311-07 | LIHC | 3.45070843 | 0.658375817 | 2.011213533 |
| TCGA-BC-A69H-01A-11R-A311-07 | LIHC | 3.539169537 | 0.658527283 | 3.592387436 |
| TCGA-DD-AAD0-01A-11R-A41C-07 | LIHC | 3.345076209 | 0.658686992 | 3.661370418 |
| TCGA-DD-AAVZ-01A-11R-A41C-07 | LIHC | 3.551939984 | 0.658782491 | 3.895719226 |
| TCGA-DD-AACD-01A-11R-A41C-07 | LIHC | 3.425818347 | 0.658991922 | 1.810754499 |
| TCGA-RC-A7SB-01A-21R-A352-07 | LIHC | 3.318605436 | 0.659087493 | 2.069129554 |
| TCGA-CC-A8HU-01A-11R-A36F-07 | LIHC | 3.417863073 | 0.659313003 | 4.851805697 |
| TCGA-DD-A39Z-01A-11R-A213-07 | LIHC | 3.388008792 | 0.659390176 | 2.126654768 |
| TCGA-BC-4073-01B-02R-A131-07 | LIHC | 3.633379258 | 0.660067918 | 3.992863581 |
| TCGA-ZP-A9CV-01A-11R-A38B-07 | LIHC | 3.490558349 | 0.660098083 | 3.660716532 |
| TCGA-ED-A7PZ-01A-11R-A33R-07 | LIHC | 3.251456832 | 0.660116149 | 3.625301756 |
| TCGA-BD-A3ER-01A-11R-A213-07 | LIHC | 3.447133531 | 0.660664528 | 1.341629473 |
| TCGA-BW-A5NO-01A-11R-A27V-07 | LIHC | 3.395565162 | 0.660720508 | 3.090887995 |
| TCGA-DD-AADM-01A-11R-A41C-07 | LIHC | 3.56154737 | 0.661231507 | 2.675083167 |
| TCGA-G3-A25X-01A-11R-A16W-07 | LIHC | 3.656836673 | 0.661254949 | 3.159943265 |
| TCGA-DD-AADN-01A-11R-A41C-07 | LIHC | 3.550440323 | 0.661344643 | 3.246548836 |
| TCGA-UB-A7MF-01A-11R-A33J-07 | LIHC | 3.468419343 | 0.661413133 | 4.184648677 |
| TCGA-DD-AACX-01A-11R-A41C-07 | LIHC | 3.396117934 | 0.661543006 | 3.087924228 |
| TCGA-G3-A7M7-01A-12R-A352-07 | LIHC | 3.368868003 | 0.66163635 | 2.144160367 |
| TCGA-DD-A39X-01A-11R-A213-07 | LIHC | 3.456390117 | 0.661698289 | 2.34008416 |
| TCGA-DD-AAVY-01A-11R-A41C-07 | LIHC | 3.256695259 | 0.661893387 | 1.323221766 |
| TCGA-RC-A6M4-01A-11R-A32O-07 | LIHC | 3.335139234 | 0.661942868 | 1.925026931 |
| TCGA-XR-A8TE-01A-11R-A36F-07 | LIHC | 3.38923937 | 0.662481548 | 0.928514917 |
| TCGA-2Y-A9H7-01A-11R-A39D-07 | LIHC | 3.605287049 | 0.662515363 | 2.595381548 |
| TCGA-KR-A7K7-01A-11R-A33J-07 | LIHC | 3.369323704 | 0.662623257 | 4.006507113 |
| TCGA-DD-AAD6-01A-11R-A41C-07 | LIHC | 3.388174612 | 0.662832 | 2.541688502 |
| TCGA-5R-AA1D-01A-11R-A38B-07 | LIHC | 3.581523878 | 0.662890605 | 1.13848843 |
| TCGA-DD-A114-01A-11R-A131-07 | LIHC | 3.775997379 | 0.66291277 | 4.291001657 |
| TCGA-G3-A3CK-01A-11R-A213-07 | LIHC | 3.358577887 | 0.663091395 | 2.313258717 |
| TCGA-LG-A9QD-01A-11R-A38B-07 | LIHC | 3.322301538 | 0.663124145 | 1.275217683 |
| TCGA-G3-A25Y-01A-11R-A16W-07 | LIHC | 3.654935216 | 0.663196099 | 4.433390402 |
| TCGA-G3-AAV4-01A-11R-A38B-07 | LIHC | 3.516058998 | 0.663579295 | 3.263875101 |
| TCGA-DD-A73B-01A-12R-A32O-07 | LIHC | 3.493629011 | 0.663688431 | 3.442874927 |
| TCGA-PD-A5DF-01A-11R-A27V-07 | LIHC | 3.628187649 | 0.66394581 | 2.874070939 |
| TCGA-5R-AA1C-01A-11R-A41C-07 | LIHC | 3.509715153 | 0.664029281 | 2.882897288 |
| TCGA-DD-AACV-01A-11R-A41C-07 | LIHC | 3.370428335 | 0.664054053 | 4.085020379 |
| TCGA-DD-AAC8-01A-11R-A41C-07 | LIHC | 3.373473215 | 0.664333847 | 2.870664537 |
| TCGA-DD-AADB-01A-11R-A41C-07 | LIHC | 3.613351353 | 0.664544333 | 3.777096132 |
| TCGA-ED-A459-01A-11R-A266-07 | LIHC | 3.560197107 | 0.66503152 | 3.733847885 |
| TCGA-DD-AACA-02B-11R-A41C-07 | LIHC | 3.383327182 | 0.665135489 | 2.647481393 |
| TCGA-DD-AADD-01A-11R-A41C-07 | LIHC | 3.551668966 | 0.665254576 | 4.339296653 |
| TCGA-G3-A25U-01A-11R-A16W-07 | LIHC | 3.327475713 | 0.66589735 | 2.782896674 |
| TCGA-GJ-A9DB-01A-11R-A37K-07 | LIHC | 3.347432791 | 0.665909536 | 3.156262751 |
| TCGA-CC-A9FW-01A-11R-A37K-07 | LIHC | 3.387088992 | 0.666438052 | 3.688888687 |
| TCGA-DD-A1EK-01A-11R-A213-07 | LIHC | 3.439376375 | 0.666616719 | 1.645461804 |
| TCGA-G3-AAV5-01A-11R-A37K-07 | LIHC | 3.320036698 | 0.667047618 | 2.340043467 |
| TCGA-DD-AAEG-01A-11R-A39D-07 | LIHC | 3.399824491 | 0.667119122 | 2.926873077 |
| TCGA-DD-AAD2-01A-11R-A41C-07 | LIHC | 3.606969666 | 0.667131756 | 2.829373397 |
| TCGA-FV-A2QQ-01A-11R-A22L-07 | LIHC | 3.472967719 | 0.667525197 | 2.471101469 |
| TCGA-CC-5259-01A-31R-A213-07 | LIHC | 3.480176389 | 0.667920125 | 2.535431368 |
| TCGA-G3-A5SM-01A-12R-A28V-07 | LIHC | 3.445190872 | 0.667945152 | 3.641690907 |
| TCGA-DD-AAVU-01A-11R-A41C-07 | LIHC | 3.385454877 | 0.668863134 | 2.338695162 |
| TCGA-2Y-A9GY-01A-11R-A38B-07 | LIHC | 3.565875202 | 0.668969306 | 3.399101586 |
| TCGA-XR-A8TD-01A-12R-A39D-07 | LIHC | 3.557390395 | 0.669059086 | 3.752806371 |
| TCGA-CC-A7II-01A-11R-A33J-07 | LIHC | 3.691576828 | 0.669832227 | 4.997777683 |
| TCGA-DD-AACZ-01A-11R-A41C-07 | LIHC | 3.768143914 | 0.669920653 | 3.587144421 |
| TCGA-DD-A39V-01A-11R-A213-07 | LIHC | 3.58613369 | 0.67035916 | 1.77362504 |
| TCGA-DD-AADJ-01A-11R-A41C-07 | LIHC | 3.575274016 | 0.670458612 | 3.220525092 |
| TCGA-2Y-A9GS-01A-12R-A38B-07 | LIHC | 3.594810826 | 0.670692946 | 3.937707639 |
| TCGA-DD-A1EI-01A-11R-A131-07 | LIHC | 3.615809339 | 0.670984572 | 2.716931502 |
| TCGA-DD-A1EE-01A-11R-A131-07 | LIHC | 3.382249652 | 0.671129749 | 2.518152241 |
| TCGA-BC-A10Z-01A-11R-A131-07 | LIHC | 3.300219567 | 0.671519947 | 3.072869132 |
| TCGA-DD-A118-01A-11R-A131-07 | LIHC | 3.491609287 | 0.672684581 | 4.515965699 |
| TCGA-DD-AADR-01A-11R-A41C-07 | LIHC | 3.590351908 | 0.673145351 | 2.398265927 |
| TCGA-G3-AAV7-01A-11R-A38B-07 | LIHC | 3.798411885 | 0.673418704 | 6.02148905 |
| TCGA-2Y-A9H0-01A-11R-A38B-07 | LIHC | 3.553833867 | 0.673576155 | 3.955949637 |
| TCGA-CC-A7IE-01A-21R-A38B-07 | LIHC | 3.526892953 | 0.674616677 | 3.57840583 |
| TCGA-CC-5262-01A-01R-A131-07 | LIHC | 3.761044919 | 0.675139647 | 3.481881015 |
| TCGA-DD-AAVX-01A-11R-A41C-07 | LIHC | 3.424990666 | 0.675681043 | 1.500020412 |
| TCGA-FV-A4ZQ-01A-11R-A266-07 | LIHC | 3.665042997 | 0.675708919 | 4.728601936 |
| TCGA-DD-AADL-01A-11R-A41C-07 | LIHC | 3.361406141 | 0.676538934 | 2.444739481 |
| TCGA-DD-AAE9-01A-11R-A41C-07 | LIHC | 3.403957479 | 0.67719679 | 2.779154751 |
| TCGA-MI-A75I-01A-11R-A32O-07 | LIHC | 3.430257744 | 0.677997766 | 3.418934227 |
| TCGA-RG-A7D4-01A-12R-A33R-07 | LIHC | 3.453016281 | 0.678048368 | 3.576454631 |
| TCGA-CC-A7IJ-01A-11R-A33R-07 | LIHC | 3.928952719 | 0.678833474 | 4.970812501 |
| TCGA-CC-A8HT-01A-11R-A36F-07 | LIHC | 3.515533756 | 0.679016144 | 4.772085141 |
| TCGA-CC-A123-01A-11R-A131-07 | LIHC | 3.562710486 | 0.679210118 | 2.421174804 |
| TCGA-FV-A3I1-01A-11R-A22L-07 | LIHC | 3.553855868 | 0.679748998 | 3.160894876 |
| TCGA-G3-A5SI-01A-31R-A27V-07 | LIHC | 3.587633288 | 0.68073806 | 4.428682593 |
| TCGA-UB-A7MD-01A-12R-A352-07 | LIHC | 3.441257207 | 0.681256238 | 3.197017584 |
| TCGA-DD-A3A4-01A-11R-A22L-07 | LIHC | 3.360039048 | 0.681368788 | 3.668098446 |
| TCGA-5C-A9VG-01A-11R-A37K-07 | LIHC | 3.595023749 | 0.681468632 | 4.964226448 |
| TCGA-BC-A3KG-01A-11R-A213-07 | LIHC | 3.50625291 | 0.682125744 | 4.023783965 |
| TCGA-G3-A7M8-01A-11R-A33R-07 | LIHC | 3.386745283 | 0.683013871 | 0.897147065 |
| TCGA-DD-A73F-01A-11R-A32O-07 | LIHC | 3.597382532 | 0.683185943 | 3.973221412 |
| TCGA-DD-AAEI-01A-11R-A41C-07 | LIHC | 3.604598976 | 0.684590008 | 2.56898165 |
| TCGA-DD-AACK-01A-11R-A41C-07 | LIHC | 3.377430728 | 0.685023662 | 2.547760053 |
| TCGA-DD-A39Y-01A-11R-A213-07 | LIHC | 3.469554604 | 0.687928994 | 3.680447153 |
| TCGA-CC-A3MC-01A-11R-A22L-07 | LIHC | 3.556964225 | 0.688717705 | 4.47028626 |
| TCGA-DD-A1EG-01A-11R-A213-07 | LIHC | 3.614619518 | 0.688872985 | 2.421525848 |
| TCGA-CC-A5UC-01A-11R-A28V-07 | LIHC | 3.757889253 | 0.689152067 | 3.933832056 |
| TCGA-DD-AACH-01A-11R-A41C-07 | LIHC | 3.624554497 | 0.690201099 | 5.120162848 |
| TCGA-G3-A7M9-01A-23R-A352-07 | LIHC | 3.57868009 | 0.691563104 | 5.584833725 |
| TCGA-2Y-A9HA-01A-11R-A39D-07 | LIHC | 3.412924362 | 0.691939891 | 3.201829955 |
| TCGA-BC-A8YO-01A-11R-A37K-07 | LIHC | 3.488637177 | 0.692742028 | 4.32985476 |
| TCGA-CC-A1HT-01A-11R-A131-07 | LIHC | 3.79415971 | 0.692973296 | 4.95261442 |
| TCGA-DD-A4NQ-01A-21R-A28V-07 | LIHC | 3.403616791 | 0.694115012 | 4.856788371 |
| TCGA-CC-A7IK-01A-12R-A33R-07 | LIHC | 3.455592409 | 0.695402848 | 3.548310279 |
| TCGA-BC-A112-01A-11R-A131-07 | LIHC | 3.6679518 | 0.695758866 | 2.967485975 |
| TCGA-BC-A10T-01A-11R-A131-07 | LIHC | 3.562092151 | 0.697427703 | 2.295163021 |
| TCGA-DD-A39W-01A-11R-A213-07 | LIHC | 3.544896989 | 0.698427821 | 1.317963451 |
| TCGA-CC-5263-01A-01R-A131-07 | LIHC | 3.666835543 | 0.699663334 | 4.821532329 |
| TCGA-DD-AACL-01A-11R-A41C-07 | LIHC | 3.767672586 | 0.701187547 | 4.065349516 |
| TCGA-CC-5258-01A-01R-A131-07 | LIHC | 3.857616601 | 0.701563097 | 4.034978174 |
| TCGA-DD-A1EJ-01A-11R-A155-07 | LIHC | 3.622358915 | 0.701678369 | 3.380144099 |
| TCGA-QA-A7B7-01A-11R-A32O-07 | LIHC | 3.318723314 | 0.704416885 | 4.267613875 |
| TCGA-CC-A3M9-01A-11R-A213-07 | LIHC | 4.034230499 | 0.704605346 | 6.487460853 |
| TCGA-DD-AACP-01A-11R-A41C-07 | LIHC | 3.701224299 | 0.709265993 | 4.685897775 |
| TCGA-BC-A10W-01A-11R-A131-07 | LIHC | 3.760773052 | 0.725200044 | 4.942699915 |
| TCGA-DD-A1EL-01A-11R-A155-07 | LIHC | 3.609988319 | 0.736533559 | 4.022404884 |
| DO23532 | LIRI | -0.17300113 | 0.052158684 | 1.14325459 |
| DO23551 | LIRI | 0.063851607 | 0.075165253 | 0.787245818 |
| DO23546 | LIRI | 0.140909055 | 0.086200195 | 2.690227782 |
| DO50800 | LIRI | 0.236505674 | 0.087449769 | 0.947263941 |
| DO23534 | LIRI | 0.003298479 | 0.088179592 | 2.554948944 |
| DO48693 | LIRI | 0.144323101 | 0.092212095 | 2.491683286 |
| DO50808 | LIRI | 0.195524015 | 0.092249184 | 1.491044122 |
| DO45167 | LIRI | -0.197621079 | 0.092862502 | 1.360238774 |
| DO45149 | LIRI | -0.129069667 | 0.09417362 | 1.623030808 |
| DO23541 | LIRI | 0.23090223 | 0.094593107 | 3.080177715 |
| DO23540 | LIRI | -0.034007308 | 0.095003457 | 2.004345649 |
| DO45249 | LIRI | 0.060773095 | 0.097022518 | 1.522181334 |
| DO50819 | LIRI | 0.315840482 | 0.098659996 | 2.984990978 |
| DO45185 | LIRI | 0.372355163 | 0.099005129 | 2.6924767 |
| DO45281 | LIRI | -0.145169096 | 0.099792695 | 0.870219645 |
| DO23547 | LIRI | 0.17984335 | 0.102321016 | 1.893749824 |
| DO45107 | LIRI | -0.150248605 | 0.103023436 | 1.218671596 |
| DO45273 | LIRI | 0.248368841 | 0.103073134 | 3.072912317 |
| DO48721 | LIRI | 0.035833003 | 0.103435552 | 2.788484069 |
| DO45103 | LIRI | 0.058140494 | 0.105317288 | 2.294279667 |
| DO50845 | LIRI | -0.130874982 | 0.105686387 | 2.091589626 |
| DO45109 | LIRI | -0.009566323 | 0.105705011 | 0.952045692 |
| DO48732 | LIRI | 0.352122645 | 0.10701032 | 3.291954674 |
| DO48719 | LIRI | 0.070208606 | 0.10708698 | 1.687132392 |
| DO45215 | LIRI | -0.158704525 | 0.108027467 | 0.882238404 |
| DO23509 | LIRI | 0.246144378 | 0.109048726 | 4.051512385 |
| DO45141 | LIRI | 0.136354718 | 0.110575673 | 2.239503697 |
| DO45241 | LIRI | -0.046174485 | 0.110661254 | 1.588254694 |
| DO45101 | LIRI | -0.115002346 | 0.111060215 | 1.2312397 |
| DO23523 | LIRI | 0.248641854 | 0.111737965 | 1.247752554 |
| DO45277 | LIRI | 0.388371586 | 0.113051521 | 2.171761166 |
| DO23535 | LIRI | 0.187211853 | 0.113118122 | 2.228945285 |
| DO50857 | LIRI | 0.172169239 | 0.11327992 | 3.38029911 |
| DO23512 | LIRI | -0.133922986 | 0.113318752 | 2.244532721 |
| DO50817 | LIRI | -0.157498202 | 0.113480817 | 2.518606228 |
| DO50774 | LIRI | 0.259085877 | 0.114227682 | 2.415146629 |
| DO48701 | LIRI | 0.157454033 | 0.114400433 | 2.333122333 |
| DO45105 | LIRI | 0.001672179 | 0.11511143 | 3.136555456 |
| DO45243 | LIRI | 0.047670365 | 0.115538329 | 1.417037538 |
| DO45201 | LIRI | 0.067684727 | 0.115964042 | 0.856272828 |
| DO45301 | LIRI | 0.058099367 | 0.115973267 | 4.264110068 |
| DO50814 | LIRI | -0.082068964 | 0.116058161 | 1.572336266 |
| DO45195 | LIRI | -0.102837684 | 0.116514663 | 0.509011737 |
| DO48672 | LIRI | 0.172840205 | 0.117089308 | 2.047759223 |
| DO23526 | LIRI | 0.204947394 | 0.119225219 | 2.932541788 |
| DO45111 | LIRI | 0.065674867 | 0.119550376 | 2.769954498 |
| DO227643 | LIRI | 0.010036425 | 0.119898135 | 1.482999409 |
| DO45289 | LIRI | 0.05766091 | 0.120092698 | 1.947041174 |
| DO48674 | LIRI | -0.178385 | 0.120323484 | 0.407983476 |
| DO50816 | LIRI | -0.115776836 | 0.120885105 | 1.381266691 |
| DO48736 | LIRI | 0.146114383 | 0.121253829 | 1.383764712 |
| DO50798 | LIRI | 0.177050091 | 0.12172438 | 3.572364272 |
| DO45179 | LIRI | 0.160118187 | 0.122272329 | 2.171062773 |
| DO23517 | LIRI | -0.09095464 | 0.122835398 | 0.457578552 |
| DO50787 | LIRI | 0.283971996 | 0.123038091 | 1.856102179 |
| DO50839 | LIRI | -0.145001478 | 0.123194785 | 0.433298258 |
| DO45096 | LIRI | 0.382163417 | 0.123249185 | 3.770036881 |
| DO50832 | LIRI | 0.150378903 | 0.123308437 | 4.841070343 |
| DO50811 | LIRI | -0.066609181 | 0.123443307 | 1.619155424 |
| DO50804 | LIRI | 0.059666866 | 0.123737168 | 1.334977053 |
| DO50807 | LIRI | 0.32873029 | 0.124007321 | 2.893991872 |
| DO50840 | LIRI | 0.19946418 | 0.124112039 | 3.521420555 |
| DO48746 | LIRI | 0.02017314 | 0.124449432 | 1.963069376 |
| DO23531 | LIRI | 0.124422279 | 0.124499019 | 0.743585595 |
| DO45197 | LIRI | 0.344894653 | 0.124853327 | 5.419196449 |
| DO45223 | LIRI | 0.232947134 | 0.12531161 | 2.2088577 |
| DO45095 | LIRI | 0.05863867 | 0.125972895 | 1.856916527 |
| DO23539 | LIRI | 0.137792528 | 0.125982577 | 3.490953292 |
| DO45263 | LIRI | 0.51290207 | 0.126001951 | 3.710239155 |
| DO50855 | LIRI | 0.060541839 | 0.126430097 | 2.02224863 |
| DO45285 | LIRI | 0.246874991 | 0.126619214 | 1.518143964 |
| DO23527 | LIRI | 0.195563474 | 0.127600201 | 1.635499058 |
| DO45233 | LIRI | 0.168891023 | 0.127693804 | 1.383574079 |
| DO50803 | LIRI | 0.22170031 | 0.127809423 | 1.780084897 |
| DO45207 | LIRI | 0.264474199 | 0.127812216 | 3.364601697 |
| DO45251 | LIRI | 0.017269454 | 0.12821264 | 1.601660304 |
| DO23518 | LIRI | 0.168145232 | 0.129179586 | 3.653559385 |
| DO45165 | LIRI | 0.344121074 | 0.12921167 | 2.958705435 |
| DO45287 | LIRI | 0.484826922 | 0.12964953 | 3.297064318 |
| DO23511 | LIRI | -0.146678338 | 0.129783904 | 2.013006266 |
| DO50805 | LIRI | 0.12559619 | 0.129914881 | 1.50533523 |
| DO23549 | LIRI | 0.312572949 | 0.130008676 | 3.180963887 |
| DO50776 | LIRI | 0.048952884 | 0.131151918 | 1.577957952 |
| DO23543 | LIRI | 0.263269754 | 0.131331984 | 3.398043877 |
| DO23548 | LIRI | 0.037277697 | 0.131836783 | 2.233325225 |
| DO48697 | LIRI | 0.297183145 | 0.132132321 | 2.813427357 |
| DO45145 | LIRI | 0.581341791 | 0.132305238 | 4.721040648 |
| DO45181 | LIRI | 0.145970337 | 0.132573827 | 0.715459308 |
| DO50813 | LIRI | -0.042138015 | 0.132606869 | 1.359841428 |
| DO48733 | LIRI | 0.219313437 | 0.132746172 | 1.69095074 |
| DO48677 | LIRI | -0.072721113 | 0.133410579 | 1.276611974 |
| DO50815 | LIRI | 0.505926439 | 0.133440967 | 4.445961337 |
| DO50850 | LIRI | 0.216980299 | 0.133690553 | 1.949551172 |
| DO45125 | LIRI | -0.014168759 | 0.133845995 | 2.745376614 |
| DO23536 | LIRI | 0.202466464 | 0.134108731 | 1.487033221 |
| DO45231 | LIRI | 0.356194219 | 0.134126595 | 2.782948328 |
| DO50793 | LIRI | -0.003020835 | 0.134849806 | 2.003242706 |
| DO45279 | LIRI | 0.119202041 | 0.135120629 | 0.934218302 |
| DO50778 | LIRI | 0.578967272 | 0.135204262 | 3.745076895 |
| DO45094 | LIRI | 0.064228751 | 0.135361776 | 1.195727453 |
| DO23521 | LIRI | 0.438249835 | 0.135396124 | 4.097873378 |
| DO45173 | LIRI | 0.412060196 | 0.135401381 | 2.305965269 |
| DO48695 | LIRI | 0.175228816 | 0.135766304 | 1.636684606 |
| DO50789 | LIRI | 0.130946928 | 0.135804715 | 2.80783072 |
| DO48728 | LIRI | 0.380217269 | 0.136477003 | 3.515186036 |
| DO48716 | LIRI | 0.108592661 | 0.13651586 | 2.260600439 |
| DO48759 | LIRI | 0.124202218 | 0.136675527 | 2.301842398 |
| DO48742 | LIRI | 0.131672269 | 0.136869506 | 0.894848374 |
| DO48725 | LIRI | 0.280050518 | 0.136997161 | 1.928275823 |
| DO45117 | LIRI | 0.320346926 | 0.138129238 | 4.95491328 |
| DO23545 | LIRI | 0.308533187 | 0.138156645 | 3.146331234 |
| DO23530 | LIRI | 0.360486734 | 0.138426738 | 2.222480417 |
| DO23544 | LIRI | -0.100199586 | 0.13862275 | 0.847238136 |
| DO23550 | LIRI | 0.18525076 | 0.138990837 | 1.889937584 |
| DO48679 | LIRI | 0.242812236 | 0.139242777 | 2.160502985 |
| DO45227 | LIRI | 0.277096723 | 0.139786729 | 2.342509328 |
| DO50818 | LIRI | 0.157667813 | 0.140041762 | 1.413964122 |
| DO45139 | LIRI | 0.109825874 | 0.140063873 | 2.642255441 |
| DO48715 | LIRI | 0.220404532 | 0.140075299 | 2.501817444 |
| DO23552 | LIRI | 0.271864022 | 0.140247747 | 5.1381521 |
| DO48700 | LIRI | 0.180636341 | 0.140299056 | 2.300781485 |
| DO45225 | LIRI | 0.617957329 | 0.140403484 | 3.906299769 |
| DO45133 | LIRI | 0.060866305 | 0.140648955 | 3.745230504 |
| DO48720 | LIRI | 0.537930976 | 0.141709396 | 3.135520463 |
| DO45137 | LIRI | 0.229176891 | 0.142056223 | 2.323976299 |
| DO50844 | LIRI | 0.235026405 | 0.142181789 | 2.74276586 |
| DO23538 | LIRI | 0.220480655 | 0.142200752 | 2.051729087 |
| DO45155 | LIRI | 0.660061528 | 0.1422898 | 3.209095196 |
| DO45229 | LIRI | 0.169032188 | 0.142357304 | 1.810927628 |
| DO48761 | LIRI | 0.186417539 | 0.142360919 | 2.202436787 |
| DO48706 | LIRI | 0.243642949 | 0.142589766 | 2.018645418 |
| DO50783 | LIRI | 0.023026164 | 0.142788007 | 1.179960543 |
| DO45097 | LIRI | 0.011896451 | 0.142934861 | 1.615220952 |
| DO45143 | LIRI | 0.141508811 | 0.143974233 | 1.651277261 |
| DO45205 | LIRI | 0.300363125 | 0.144510402 | 2.930551542 |
| DO23510 | LIRI | 0.229696528 | 0.144801544 | 2.201335544 |
| DO45177 | LIRI | 0.233125978 | 0.14516229 | 1.788867327 |
| DO45203 | LIRI | 0.357978748 | 0.145245754 | 3.006477067 |
| DO45159 | LIRI | 0.239003607 | 0.14531589 | 2.898236093 |
| DO45283 | LIRI | 0.274036796 | 0.145317896 | 2.3762056 |
| DO227801 | LIRI | 0.180357374 | 0.145636635 | 1.540601071 |
| DO45255 | LIRI | 0.116792016 | 0.145751712 | 3.109670611 |
| DO50806 | LIRI | 0.257579408 | 0.145823043 | 2.859677319 |
| DO45121 | LIRI | 0.194596159 | 0.146110652 | 2.577991303 |
| DO23528 | LIRI | 0.050813981 | 0.146420767 | 3.26629034 |
| DO45237 | LIRI | 0.568500728 | 0.146602489 | 5.2616689 |
| DO45153 | LIRI | 0.264403605 | 0.147399727 | 2.908796499 |
| DO45247 | LIRI | 0.311740556 | 0.147695171 | 2.159871412 |
| DO45305 | LIRI | 0.14513182 | 0.148411716 | 2.090408251 |
| DO48760 | LIRI | 0.135043544 | 0.148503793 | 1.499216268 |
| DO48717 | LIRI | 0.337263934 | 0.1499985 | 3.409524037 |
| DO45135 | LIRI | 0.453238283 | 0.150293647 | 2.147711467 |
| DO45303 | LIRI | 0.280638958 | 0.150855291 | 2.429126276 |
| DO45219 | LIRI | 0.288574021 | 0.150856348 | 2.945339562 |
| DO23533 | LIRI | 0.071817359 | 0.15104429 | 3.087486836 |
| DO45189 | LIRI | 0.436695943 | 0.151351084 | 2.599422086 |
| DO45115 | LIRI | 0.267969704 | 0.151583844 | 2.69329422 |
| DO48712 | LIRI | 0.332110583 | 0.152010198 | 2.812958937 |
| DO23537 | LIRI | 0.363213268 | 0.152349153 | 4.174891552 |
| DO45127 | LIRI | 0.802378921 | 0.152876977 | 5.405120799 |
| DO45099 | LIRI | 0.115195814 | 0.152878679 | 2.390030649 |
| DO48757 | LIRI | 0.293768928 | 0.153208636 | 3.386746731 |
| DO45187 | LIRI | 0.41298871 | 0.153230813 | 3.371922821 |
| DO45245 | LIRI | 0.293519887 | 0.153471354 | 3.837530542 |
| DO45119 | LIRI | 0.419904942 | 0.153525235 | 2.946499378 |
| DO50809 | LIRI | 0.246580108 | 0.153641324 | 3.396157344 |
| DO48737 | LIRI | 0.183594295 | 0.153926192 | 2.635577106 |
| DO23515 | LIRI | 0.419783428 | 0.154199568 | 3.665875386 |
| DO48747 | LIRI | 0.437754805 | 0.154700327 | 2.574479534 |
| DO45129 | LIRI | 0.115447339 | 0.154904104 | 2.884253893 |
| DO45131 | LIRI | 0.258245305 | 0.156174473 | 3.107550495 |
| DO45261 | LIRI | 0.258374407 | 0.157223258 | 1.757773627 |
| DO50859 | LIRI | 0.018778164 | 0.157540086 | 1.540918066 |
| DO48704 | LIRI | 0.116958923 | 0.157743086 | 2.758609741 |
| DO48682 | LIRI | 0.157676923 | 0.158007804 | 3.370957284 |
| DO50825 | LIRI | 0.23152808 | 0.15806929 | 2.459315676 |
| DO45259 | LIRI | 0.399717359 | 0.158149881 | 3.389188302 |
| DO23529 | LIRI | 0.140683953 | 0.159789796 | 3.006203835 |
| DO48743 | LIRI | 0.236158291 | 0.160266692 | 2.647778963 |
| DO45297 | LIRI | 0.169024291 | 0.16045527 | 2.872203595 |
| DO45191 | LIRI | 0.074065226 | 0.160955422 | 1.816340037 |
| DO45091 | LIRI | 0.278563692 | 0.162531053 | 4.869135374 |
| DO23516 | LIRI | 0.04219419 | 0.163474371 | 3.945767862 |
| DO48751 | LIRI | 0.098514829 | 0.163728763 | 2.263795694 |
| DO45183 | LIRI | 0.284248387 | 0.163866117 | 1.930773555 |
| DO45171 | LIRI | 0.105200526 | 0.164017241 | 1.821499143 |
| DO45175 | LIRI | 0.103193561 | 0.164582011 | 3.891753669 |
| DO45157 | LIRI | 0.464749372 | 0.165101749 | 2.958446569 |
| DO48741 | LIRI | 0.243389795 | 0.165650777 | 2.644555065 |
| DO48727 | LIRI | 0.09082127 | 0.165857736 | 1.512613487 |
| DO45163 | LIRI | 0.156840534 | 0.166190027 | 2.176094397 |
| DO23524 | LIRI | 0.545738558 | 0.166509392 | 3.582017247 |
| DO45199 | LIRI | 0.175736822 | 0.166681835 | 1.916657841 |
| DO45169 | LIRI | 0.154677922 | 0.166695298 | 2.814667331 |
| DO45161 | LIRI | 0.451288831 | 0.166974203 | 3.380347443 |
| DO50785 | LIRI | 0.149730339 | 0.167125986 | 2.404273574 |
| DO50820 | LIRI | 0.386717636 | 0.167926551 | 3.527289088 |
| DO23514 | LIRI | 0.497169633 | 0.167956808 | 3.05778659 |
| DO23525 | LIRI | 0.391339167 | 0.168619284 | 3.629813747 |
| DO45217 | LIRI | 0.180153196 | 0.168723402 | 3.89863266 |
| DO45123 | LIRI | 0.143196484 | 0.16876649 | 3.099289947 |
| DO23513 | LIRI | 0.292343939 | 0.169028323 | 6.014932339 |
| DO48681 | LIRI | 0.493312282 | 0.169143516 | 3.511812377 |
| DO45253 | LIRI | 0.240278341 | 0.170247205 | 3.584154617 |
| DO45275 | LIRI | 0.607019975 | 0.172107299 | 3.909812563 |
| DO45239 | LIRI | 0.409604578 | 0.172123075 | 2.653997997 |
| DO45211 | LIRI | 0.563022673 | 0.173088502 | 3.735262415 |
| DO45235 | LIRI | 0.311080695 | 0.173362415 | 3.032480383 |
| DO50799 | LIRI | 0.408680128 | 0.173883438 | 3.484304334 |
| DO45299 | LIRI | 0.336702722 | 0.174060081 | 3.740461584 |
| DO50802 | LIRI | 0.05767112 | 0.176991634 | 1.697832281 |
| DO50822 | LIRI | 0.282573122 | 0.178439969 | 1.851418252 |
| DO45209 | LIRI | 0.260748283 | 0.17968827 | 3.766677874 |
| DO45307 | LIRI | 0.495996807 | 0.180515959 | 3.531324696 |
| DO45092 | LIRI | 0.320885182 | 0.181279806 | 2.734677603 |
| DO48730 | LIRI | 0.429168066 | 0.182965788 | 2.986243081 |
| DO45265 | LIRI | 0.440942231 | 0.184301202 | 2.396400568 |
| DO45113 | LIRI | 0.238081846 | 0.184635548 | 3.179112084 |
| DO23519 | LIRI | 0.447728672 | 0.184745876 | 3.88378527 |
| DO45213 | LIRI | 0.342929696 | 0.185094714 | 3.46581057 |
| DO45193 | LIRI | 0.397857275 | 0.185828318 | 3.793876776 |
| DO50829 | LIRI | 0.515457633 | 0.188062265 | 3.675370551 |
| DO23542 | LIRI | 0.400465286 | 0.188160424 | 2.793446168 |
| DO45257 | LIRI | 0.246454141 | 0.188328469 | 5.365537598 |
| DO45093 | LIRI | 0.296640271 | 0.189481447 | 3.601063891 |
| DO45267 | LIRI | 0.268997818 | 0.191565293 | 3.734196691 |
| DO48723 | LIRI | 0.334351305 | 0.192929937 | 3.088284388 |
| DO45221 | LIRI | 0.444036387 | 0.193552884 | 2.853084566 |
| DO50791 | LIRI | 0.325369164 | 0.195452177 | 3.637487606 |
| DO45269 | LIRI | 0.355381248 | 0.200406993 | 3.659852585 |
| DO23508 | LIRI | 0.359876596 | 0.201686628 | 2.935936551 |

| **Table S2 The correlation between metabolisms and stemnesss index or AUKRA expression in HCC.** |  |  |  |  |
| --- | --- | --- | --- | --- |
| **Metabolism processes** | **Stemness index** | | **AURKA expression** | |
|  | **PCC** | **adj.**p.val | **PCC** | **adj.**p.val |
| ADP-Ribosylation | 0.10 | 0.075398429 | -0.27 | 4.11E-07 |
| Alanine, Aspartate and Glutamate Metabolism | -0.13 | 0.019093503 | -0.24 | 7.80E-06 |
| Aldosterone Biosynthesis | -0.26 | 9.17E-07 | -0.19 | 0.000345582 |
| alpha-Linoleic Acid Metabolism | 0.06 | 0.308492014 | -0.11 | 0.05440352 |
| Amino Sugar and Nucleotide Sugar Metabolism | 0.32 | 8.59E-10 | -0.06 | 0.320080366 |
| Arachidonic Acid Metabolism | 0.08 | 0.158396425 | -0.24 | 5.44E-06 |
| Arginine and Proline Metabolism | -0.17 | 0.001978966 | -0.25 | 3.81E-06 |
| Arginine Biosynthesis | -0.27 | 4.51E-07 | -0.26 | 1.15E-06 |
| Ascorbate and Aldrate Metabolism | -0.15 | 0.005456025 | -0.14 | 0.012981042 |
| Beta-Alanine Metabolism | -0.34 | 1.97E-10 | -0.45 | 8.66E-18 |
| Biosynthesis of Unsaturated Fatty Acids | 0.03 | 0.540717894 | -0.13 | 0.015392547 |
| Biotin Metabolism | -0.28 | 1.17E-07 | -0.30 | 1.67E-08 |
| Butanoate Metabolism | -0.37 | 2.23E-12 | -0.39 | 1.31E-13 |
| Caffiene Metabolism | -0.40 | 1.25E-14 | -0.40 | 1.47E-14 |
| Cardiolipin Biosynthesis | -0.02 | 0.686815005 | -0.07 | 0.209818383 |
| Cardiolipin Metabolism | 0.07 | 0.212527912 | 0.10 | 0.090868632 |
| Cholesterol Biosynthesis | -0.04 | 0.478233934 | -0.02 | 0.759142723 |
| Citric Acid Cycle | -0.19 | 0.000390679 | -0.24 | 7.80E-06 |
| Cortisol Biosynthesis | -0.31 | 2.86E-09 | -0.22 | 4.69E-05 |
| Cyclooxygenase Arachidonic Acid Metabolism | 0.44 | 3.79E-17 | -0.07 | 0.236464735 |
| Cysteine and Methionine Metabolism | -0.17 | 0.002055197 | -0.11 | 0.046831968 |
| D-Glutamine and D-Glutamate Metabolism | -0.05 | 0.335525531 | -0.06 | 0.329443775 |
| Dopamine Biosynthesis | -0.15 | 0.006186791 | -0.13 | 0.02090606 |
| Drug Metabolism by Cytochrome P450 | -0.32 | 8.59E-10 | -0.34 | 1.36E-10 |
| Drug Metabolism by other enzymes | -0.20 | 0.000173897 | -0.22 | 4.44E-05 |
| Epinephrine Biosynthesis | -0.27 | 5.02E-07 | -0.15 | 0.008388758 |
| Estradiol Biosynthesis | -0.27 | 5.39E-07 | -0.26 | 1.48E-06 |
| Ether Lipid Metabolism | 0.36 | 1.26E-11 | 0.08 | 0.173986198 |
| Fatty Acid Biosynthesis | -0.17 | 0.001710243 | -0.16 | 0.003520921 |
| Fatty Acid Degradation | -0.39 | 3.83E-14 | -0.43 | 1.46E-16 |
| Fatty Acid Elongation | 0.22 | 3.19E-05 | 0.05 | 0.436282985 |
| Folate biosynthesis | -0.18 | 0.000974819 | -0.18 | 0.000769369 |
| Folate One Carbon Metabolism | -0.14 | 0.009698446 | -0.22 | 3.92E-05 |
| Fructose and Mannose Metabolism | 0.20 | 0.000205886 | -0.06 | 0.255164804 |
| Galactose Metabolism | 0.27 | 3.69E-07 | 0.07 | 0.199612303 |
| Gluconeogenesis | -0.25 | 3.29E-06 | -0.36 | 1.39E-11 |
| Glutathione Metabolism | 0.10 | 0.063130238 | -0.08 | 0.18309621 |
| Glycerolipid Metabolism | -0.12 | 0.032314008 | -0.22 | 6.25E-05 |
| Glycerophospholipid Metabolism | 0.14 | 0.009698446 | -0.18 | 0.000835118 |
| Glycine, Serine and Threonine Metabolism | -0.31 | 2.86E-09 | -0.27 | 3.55E-07 |
| Glycogen Biosynthesis | -0.21 | 0.000143122 | -0.20 | 0.000191471 |
| Glycogen Degradation | -0.15 | 0.00654298 | -0.24 | 1.04E-05 |
| Glycolysis | -0.17 | 0.001685724 | -0.30 | 1.67E-08 |
| Glycosaminoglycan Biosynthesis | 0.39 | 8.17E-14 | -0.12 | 0.025314877 |
| Glycosphingolipid Biosynthesis | 0.24 | 7.32E-06 | -0.14 | 0.011898451 |
| Glycosphosphatidylinositol | -0.06 | 0.274904647 | -0.02 | 0.704283119 |
| Glyoxylate and Dicarboxylate Metabolism | -0.31 | 3.40E-09 | -0.30 | 1.74E-08 |
| Heme Biosynthesis | -0.20 | 0.000173897 | -0.29 | 5.61E-08 |
| Hexosamine Biosynthesis | 0.15 | 0.004415872 | 0.09 | 0.123480349 |
| Histidine Metabolism | -0.32 | 2.06E-09 | -0.40 | 4.03E-14 |
| Homocysteine Biosynthesis | 0.06 | 0.310851821 | 0.08 | 0.173986198 |
| Inositol Phosphate Metabolism | 0.42 | 1.03E-15 | -0.01 | 0.883987919 |
| Ketone Biosynthesis and Metabolism | -0.17 | 0.002030366 | -0.23 | 2.44E-05 |
| Kynurenine Metabolism | -0.15 | 0.004975895 | -0.26 | 1.14E-06 |
| Linoleic Acid Metabolism | -0.23 | 2.66E-05 | -0.34 | 2.37E-10 |
| Lipoic Acid Metabolism | -0.16 | 0.002584052 | -0.23 | 2.21E-05 |
| Lysine Degradation | -0.19 | 0.000491486 | -0.17 | 0.001376057 |
| Metabolism of Xenobiotics by Cytochrome P450 | -0.31 | 3.92E-09 | -0.32 | 1.08E-09 |
| Methionine Cycle | 0.06 | 0.274904647 | 0.07 | 0.210753673 |
| Mucin Type O-Glycan Biosynthesis | 0.26 | 1.36E-06 | -0.09 | 0.097561845 |
| Neomycin, Kanamysin and Gentamicin Biosynthesis | 0.24 | 1.14E-05 | 0.01 | 0.891550219 |
| N-Glycan Biosynthesis | 0.25 | 2.80E-06 | -0.05 | 0.431582804 |
| Nicotinamide Adenine Dinucleotide Biosynthesis | -0.19 | 0.000343108 | -0.38 | 4.23E-13 |
| Nicotinamide Adenine Metabolism | -0.06 | 0.273943853 | -0.28 | 2.46E-07 |
| Nicotinate and Nicotinamide Metabolism | -0.01 | 0.905916255 | -0.27 | 3.35E-07 |
| Nitrogen Metabolism | -0.23 | 2.75E-05 | -0.27 | 7.26E-07 |
| Norepinephrine Biosynthesis | -0.20 | 0.000187818 | -0.14 | 0.012981042 |
| Other Glycan Degradation | -0.22 | 4.54E-05 | -0.16 | 0.004391621 |
| Other Types of O-Glycan Biosynthesis | 0.40 | 1.90E-14 | -0.01 | 0.838608256 |
| Oxidative Phosphorylation | -0.02 | 0.743733442 | -0.12 | 0.024572409 |
| Pantothenate and CoA Biosynthesis | 0.00 | 0.943114212 | -0.14 | 0.009851666 |
| Pentose and Glucuronate Interconversions | -0.11 | 0.035037822 | -0.12 | 0.026644169 |
| Pentose Phosphate | 0.17 | 0.001633986 | 0.15 | 0.008629148 |
| Phenylalanine Metabolism | -0.37 | 1.31E-12 | -0.41 | 5.92E-15 |
| Phenylalanine, Tyrosine and Tryptophan Biosynthesis | -0.36 | 3.61E-12 | -0.33 | 4.50E-10 |
| Polyamine Biosynthesis | 0.46 | 5.46E-19 | 0.13 | 0.021732879 |
| Porphyrin and Chlorophyll Metabolism | -0.13 | 0.015222923 | -0.21 | 0.000146653 |
| Primary Bile Acid Biosynthesis | -0.40 | 9.53E-15 | -0.43 | 3.54E-16 |
| Propanoate Metabolism | -0.28 | 1.52E-07 | -0.35 | 4.13E-11 |
| Prostaglandin Biosynthesis | 0.35 | 4.66E-11 | 0.04 | 0.495898001 |
| Prostanoid Biosynthesis | 0.32 | 1.05E-09 | -0.12 | 0.029476873 |
| Purine Biosynthesis | 0.51 | 1.34E-23 | 0.31 | 1.07E-08 |
| Purine Metabolism | 0.41 | 4.57E-15 | -0.01 | 0.891550219 |
| Pyrimidine Biosynthesis | 0.14 | 0.008997409 | -0.02 | 0.759142723 |
| Pyrimidine Metabolism | 0.42 | 1.03E-15 | 0.28 | 1.28E-07 |
| Pyruvate Metabolism | -0.22 | 5.29E-05 | -0.33 | 7.72E-10 |
| Remethylation | -0.20 | 0.000240929 | -0.19 | 0.000520608 |
| Retinoic Acid Metabolism | 0.07 | 0.202704472 | -0.23 | 2.00E-05 |
| Retinoid Metabolism | -0.03 | 0.569008977 | -0.20 | 0.000325368 |
| Retinol Metabolism | -0.29 | 4.62E-08 | -0.32 | 1.88E-09 |
| Riboflavin Metabolism | -0.05 | 0.408411198 | -0.02 | 0.777441862 |
| Selenocompound Metabolism | -0.16 | 0.00268754 | -0.32 | 1.50E-09 |
| Shingolipid Metabolism | 0.29 | 8.74E-08 | 0.10 | 0.060733253 |
| Sirtuin Nicotinamide Metabolism | -0.21 | 8.57E-05 | -0.16 | 0.004148468 |
| Starch and Suctose Metabolism | -0.11 | 0.040939353 | -0.25 | 2.68E-06 |
| Steroid Biosynthesis | -0.05 | 0.366888663 | -0.07 | 0.206202307 |
| Steroid Hormone Biosynthesis | -0.31 | 8.03E-09 | -0.31 | 5.22E-09 |
| Steroid Hormone Metabolism | -0.31 | 6.76E-09 | -0.30 | 1.67E-08 |
| Sulfur Metabolism | -0.19 | 0.000388096 | -0.27 | 4.52E-07 |
| Taurine and Hypotaurine Metabolism | 0.00 | 0.943114212 | -0.19 | 0.000459473 |
| Terpenoid Backbone Biosynthesis | -0.04 | 0.516684731 | -0.06 | 0.323877271 |
| Testosterone Biosynthesis | -0.40 | 1.02E-14 | -0.36 | 8.03E-12 |
| Thiamine Metabolism | -0.15 | 0.00654298 | -0.26 | 1.40E-06 |
| Thromboxane Biosynthesis | 0.41 | 7.97E-15 | -0.08 | 0.169894483 |
| Transsulfuration | -0.39 | 8.17E-14 | -0.26 | 1.14E-06 |
| Tryptophan Metabolism | -0.30 | 8.59E-09 | -0.41 | 4.15E-15 |
| Tyrosine Metabolism | -0.44 | 3.79E-17 | -0.46 | 3.00E-18 |
| Ubiquinone and other Terpenoid-Quinone Biosynthesis | -0.34 | 1.29E-10 | -0.36 | 8.03E-12 |
| Urea Cycle | -0.17 | 0.001639922 | -0.16 | 0.002930595 |
| Valine, Leucine and Isoleucine Biosynthesis | 0.12 | 0.025490947 | 0.01 | 0.830878626 |
| Valine, Leucine and Isoleucine Degradation | -0.27 | 4.38E-07 | -0.35 | 7.16E-11 |
| Vitamin B10 Metabolism | -0.15 | 0.005529914 | -0.20 | 0.000238437 |
| Vitamin B11 Metabolism | 0.01 | 0.89274444 | -0.01 | 0.913086042 |
| Vitamin B6 Metabolism | -0.23 | 2.61E-05 | -0.21 | 0.000108654 |
| Vitamin B7 Metabolism | -0.03 | 0.589167948 | 0.02 | 0.744499476 |
| Vitamin B8 Metabolism | -0.14 | 0.011315388 | -0.03 | 0.664218659 |
| Vitamin B9 Metabolism | 0.14 | 0.007796837 | 0.11 | 0.055694862 |
| Vitamin K | -0.28 | 2.33E-07 | -0.28 | 2.62E-07 |
